# Physicochemical Characterization of Liposomal Bilayer Models Loaded with *Trans*-4-(4′-hydroxy-3′-methoxyphenyl)-5-(1-iodoethyl)dihydrofuran-2-one

**DOI:** 10.3390/molecules31203756

**Published:** 2026-10-19

**Authors:** Anna Dunal, Aleksandra Włoch, Witold Gładkowski, Magdalena Domańska, Hanna Pruchnik

**Affiliations:** 1 Department of Food Chemistry and Biocatalysis, Faculty of Biotechnology and Food Sciences, Wrocław University of Environmental and Life Sciences, Norwida 25, 50-375 Wrocław, Poland; witold.gladkowski@upwr.edu.pl; 2 Department of Physics and Biophysics, Faculty of Biotechnology and Food Sciences, Wrocław University of Environmental and Life Sciences, Norwida 25, 50-375 Wrocław, Poland; aleksandra.wloch@upwr.edu.pl; 3 Institute of Environmental Engineering, Faculty of Environmental Engineering and Geodesy, Wrocław University of Environmental and Life Sciences, pl. Grunwaldzki 24, 50-363 Wrocław, Poland; magdalena.domanska@upwr.edu.pl

**Keywords:** δ-iodo-γ-lactone, DPPC/cholesterol liposomes, physicochemical properties, loading efficiency, fluorescence spectroscopy, DLS, IR

## Abstract

*Trans*-4-(4′-hydroxy-3′-methoxyphenyl)-5-(1-iodoethyl)dihydrofuran-2-one (**IL**), a vanillin-derived iodolactone with previously demonstrated biological activity, was incorporated into DPPC/cholesterol (DPPC/chol) liposomes to develop and characterize a lipid-based delivery system, with particular emphasis on the relationship between **IL** incorporation and the biophysical properties of the nanocarrier. The physicochemical stability of formulations containing 2, 5, 10, and 20 mol% **IL** was assessed using dynamic and electrophoretic light scattering (DLS/ELS), while the loading efficiency (LE%) of the highest **IL** concentration tested (20 mol%) was determined via ultracentrifugation, liquid–liquid extraction, and subsequent gas chromatography (GC) analysis. The formulation exhibited a high LE% of 87.6 ± 7.8%; however, as potential sedimentation of free **IL** cannot be ruled out, this value represents an indirect estimate of **IL** loading and remains a technical limitation of the assay. The effect of **IL** incorporation on bilayer organization was examined using three fluorescent probes, as well as attenuated total reflectance Fourier-transform infrared spectroscopy. DLS/ELS measurements demonstrated the most favorable colloidal stability for liposomes hydrated with milli-Q water and stored at 4 °C, as reflected by the maintenance of low PDI values, a relatively stable hydrodynamic diameter, and a negative zeta potential throughout the one-month storage period. Fluorescence spectroscopy demonstrated that **IL** modified the temperature-dependent organization of the DPPC/chol bilayer, with effects particularly evident at the membrane interface and, at elevated temperatures, in the hydrophobic region. Complementary ATR-FTIR analysis indicated that **IL** interacts with the polar headgroup and glycerol backbone regions without significantly disrupting acyl chain organization. Hemolytic assays further demonstrated the low membrane-damaging activity of **IL**-loaded liposomes in both crude dispersion fractions and the ultracentrifuged liposomal fraction. These results demonstrate that **IL** can be efficiently incorporated into DPPC/chol liposomes while maintaining favorable physicochemical stability and low hemolytic potential. The observed effect of the tested compound on bilayer organization without disrupting membrane integrity supports the need for further investigation of lipid formulations containing **IL**.

## 1. Introduction

Lactones constitute a broad class of cyclic compounds that occur widely in nature [1,2,3,4,5,6] and exhibit a broad range of biological properties [7,8,9,10]. They are valuable leads for the discovery of novel bioactive compounds [11] and useful scaffolds for the synthesis of biologically active derivatives [12], making them of considerable interest in medicinal and pharmaceutical research [13,14]. Synthetic lactones containing aromatic substituents have drawn significant interest, primarily due to their reported antiproliferative properties [15,16,17]. Despite their therapeutic potential, the development of lactones as drug candidates remains challenging due to their structural diversity. Their application in biological systems may be limited by unfavourable physicochemical and pharmacokinetic properties, including high lipophilicity and poor water solubility [18]. Lipid-based delivery systems, including liposomes, may therefore offer a promising approach to overcoming these limitations by improving the solubility, stability, absorption, and overall bioavailability of poorly water-soluble compounds [19,20]. In addition, liposomes can provide several important advantages, including prolonged and controlled drug release, protection of encapsulated compounds against degradation, reduced systemic toxicity, and improved pharmacokinetic profiles. Liposomal systems can facilitate the efficient delivery of bioactive compounds to their target sites while reducing their susceptibility to rapid elimination from the body, thereby potentially prolonging their therapeutic effects [21]. These properties make liposomes an attractive vehicle for improving the efficacy and safety profile of targeted therapeutics.

Liposomes are spherical vesicles composed of lipid bilayers enclosing an aqueous core. Their spontaneous formation in aqueous media is driven by the amphiphilic properties of phospholipids, yielding a structure capable of encapsulating both hydrophilic and hydrophobic compounds [22,23]. However, the successful application of liposomes as advanced drug delivery systems depends on the rational design and precise optimization of their physicochemical properties. Parameters such as vesicle size, lipid composition, surface characteristics, membrane fluidity, and encapsulation efficiency critically govern colloidal stability, drug-loading capacity, release kinetics, biodistribution, and interactions with biological barriers [21]. In this context, the physicochemical characteristics of the lipid bilayer are particularly important, as they influence liposomal stability, encapsulation efficiency, and the release of active agents [24]. Therefore, careful control over lipid composition and bilayer properties is essential for obtaining stable liposomal formulations with high encapsulation efficiency and predictable release profiles. Importantly, the incorporation of a bioactive agent into a lipid bilayer is not merely passive, as the physicochemical properties of the incorporated molecule may alter membrane organization, lipid-lipid interactions, and the overall biophysical behavior of the vesicles. Thus, comprehensive characterization of both the carrier and its interaction with the encapsulated compound is essential for the rational development of liposomal delivery systems.

In our previous studies, we identified a vanillin-derived iodolactone, namely *trans*-4-(4′-hydroxy-3′-methoxyphenyl)-5-(1-iodoethyl)dihydrofuran-2-one (**IL**), as a particularly promising candidate for anticancer drug development due to its pronounced and selective cytotoxic activity against several human and canine cancer cell lines.

Importantly, **IL** did not exhibit significant cytotoxicity against non-cancerous fibroblasts or human red blood cells (RBCs), indicating a favorable selectivity profile. In addition to its antiproliferative effects, **IL** demonstrated antioxidant and moderate anti-inflammatory properties, while spectroscopic and fluorometric studies revealed its ability to interact with the polar regions of model cell membranes and modulate their organization [25,26]. These findings are particularly relevant in the context of liposomal delivery, as the demonstrated affinity of **IL** for lipid membrane environments suggests that its incorporation into a lipid bilayer may influence both its association with the carrier and the structural organization of the membrane. Accordingly, the use of liposomes as delivery systems for **IL** may offer a strategy to improve its aqueous dispersibility and physicochemical stability while modulating its interactions with biological membranes.

However, despite its promising biological profile and demonstrated membrane activity, the incorporation of **IL** into liposomal delivery systems has not been previously investigated. In particular, the effects of **IL** incorporation on colloidal stability, its association with the lipid bilayer, membrane organization, biophysical properties, and hemolytic potential have not yet been characterized.

Therefore, in the present study, we characterized DPPC/cholesterol (DPPC/chol) liposomes loaded with 20 mol% **IL**, with particular emphasis on the relationship between **IL** incorporation and the biophysical properties of the lipid bilayer. During the design of the liposomal formulation, 20 mol% cholesterol was selected based on previously reported studies of DPPC-based liposomal systems. In particular, Cheng et al. reported that the incorporation of 20 mol% cholesterol into DPPC/DPPG lipid bilayers reduced the permeability of glycine, a small hydrophilic amino acid, by approximately fourfold compared with the cholesterol-free membrane [27]. Although the DPPC/DPPG system differs from the DPPC/chol formulation investigated in the present study, this finding indicates that cholesterol content can substantially affect the permeability of DPPC-based lipid bilayers and therefore provides a rationale for selecting 20 mol% cholesterol as the initial concentration in our formulation [27]. Other studies have also employed 20 mol% cholesterol in phospholipid-based liposomal formulations [28,29]. First, the physicochemical stability of **IL**-loaded liposomes containing different **IL** concentrations was evaluated by dynamic light scattering (DLS) and electrophoretic light scattering (ELS), considering particle size, size distribution, and zeta potential under different hydration and storage conditions. The most stable formulation was then selected for further studies, including determination of the **IL** loading efficiency of the liposomes using a newly developed gas chromatographic (GC) method. The effect of the selected formulation on lipid bilayer organization and biophysical properties was further investigated using fluorescence spectroscopy with three membrane probes and ATR-FTIR spectroscopy. Finally, the hemolytic activity of **IL**-loaded liposomes in both crude dispersion fractions and the ultracentrifuged liposomal fraction was assessed. Together, these analyses were aimed at determining whether **IL** can be efficiently loaded with DPPC/chol liposomes without compromising vesicle stability and membrane integrity, while also providing insight into the biophysical effects of **IL** on the lipid bilayer.

## 2. Results and Discussion

**Figure 1:**
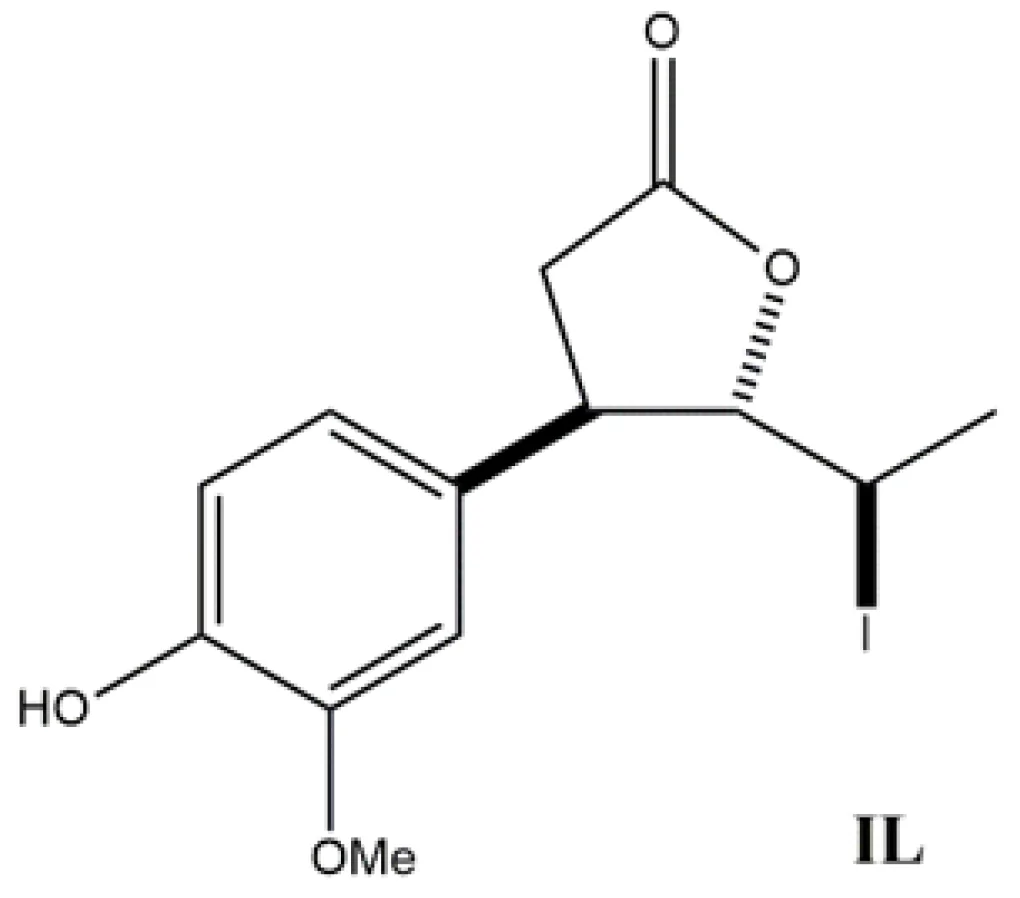
Structure of the tested iodolactone *trans*-4-(4′-hydroxy-3′-methoxyphenyl)-5-(1-iodoethyl)dihydrofuran-2-one (**IL**).

The iodolactone investigated in this study belongs to the class of β-aryl-δ-halo-γ-lactones (Figure 1). Its synthesis from vanillin and complete structural characterization were previously described in detail [25].

### 2.1. Physicochemical Characterization of Liposomes Modified with **IL**

The physicochemical properties and stability of the formed liposomes were assessed using dynamic light scattering (DLS) and electrophoretic light scattering (ELS). The analysis was performed to evaluate the effects of **IL** concentration, hydration medium (milli-Q water or PBS (phosphate buffer, pH 7.4)), and storage temperature (4 °C or room temperature (RT)) on the physicochemical stability of the formed liposomes over time. DPPC/chol liposomes without **IL** were used as a control, while **IL**-containing liposomes were prepared at concentrations of 2, 5, 10, and 20 mol% relative to the total amount of DPPC and cholesterol. The chemical structures of 1,2-dipalmitoyl-*sn*-glycero-3-phosphocholine (DPPC) and cholesterol are shown in the Supplementary Materials (Figure S1).

**Figure 2:**
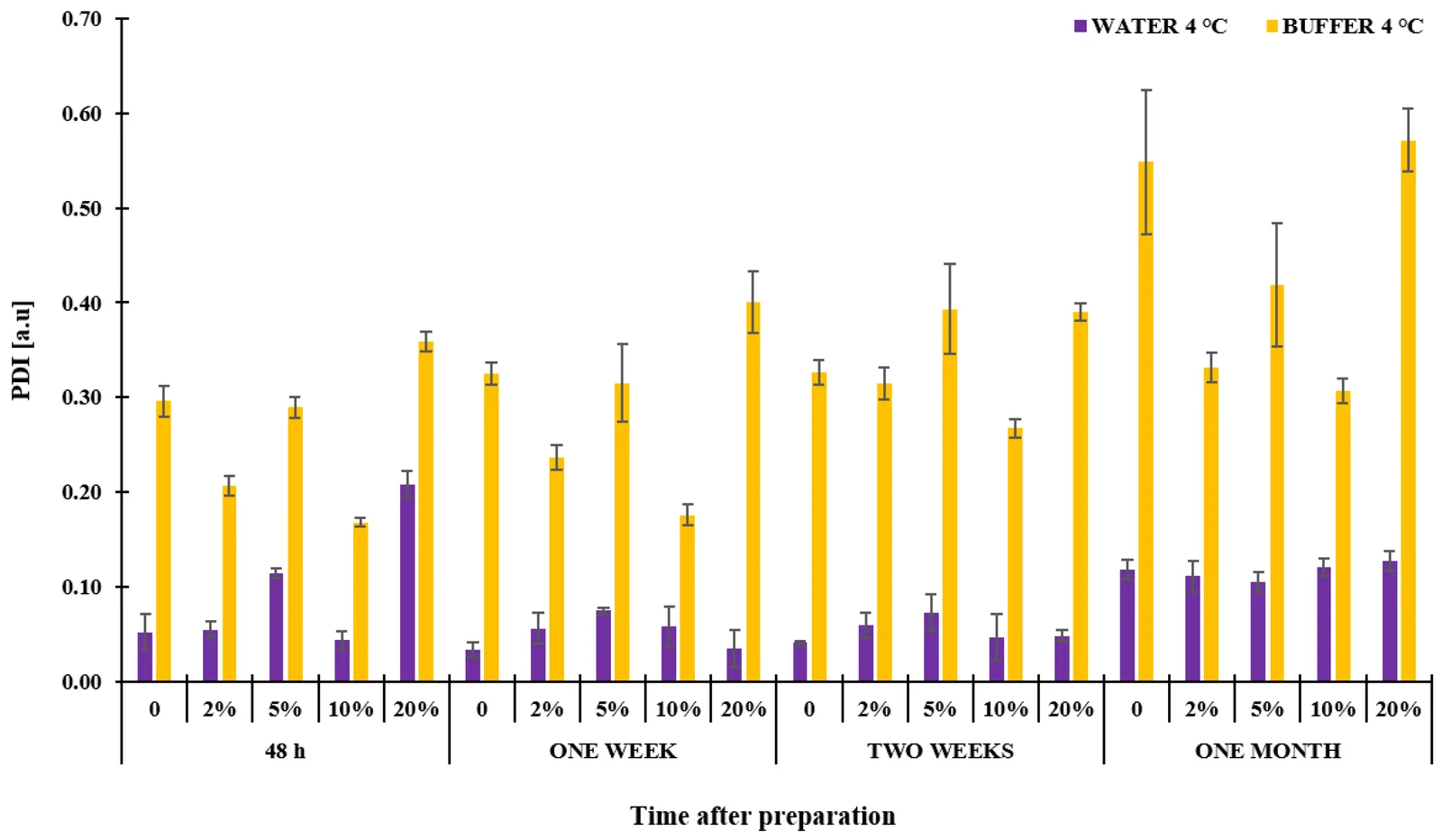
Polydispersity index (PDI) of DPPC/chol liposomes containing 0, 2, 5, 10, and 20 mol% **IL**, with a total lipid concentration of 0.13 mg/mL, measured after 48 h, one week, two weeks, and one month of storage. “0” means DPPC/chol liposomes without **IL**. Data are presented as mean ± standard deviation (*n* = 3).

**Figure 3:**
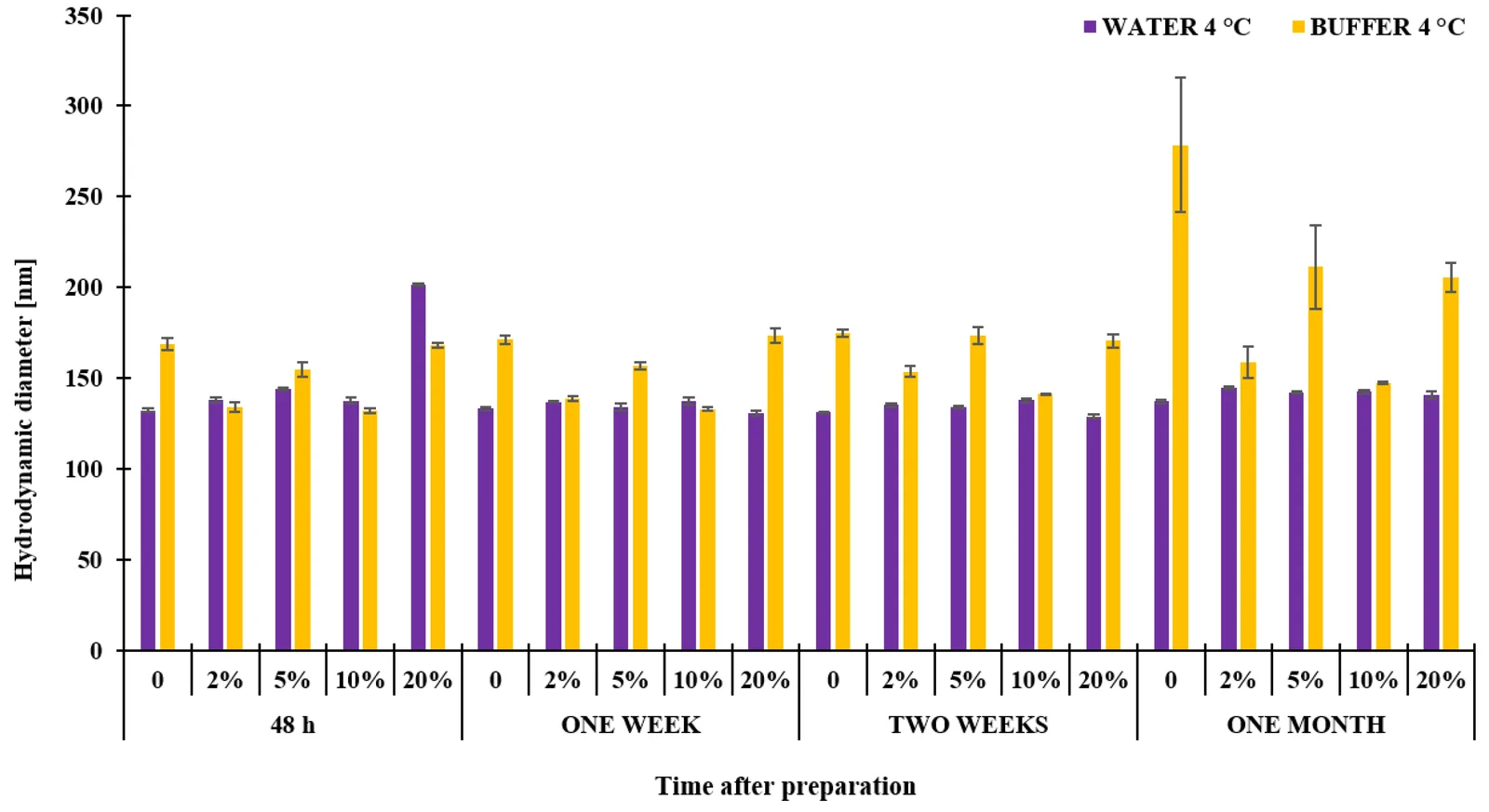
Hydrodynamic diameter of DPPC/chol liposomes containing 0, 2, 5, 10, and 20 mol% **IL**, with a total lipid concentration of 0.13 mg/mL, measured after 48 h, one week, two weeks, and one month of storage. “0” means DPPC/chol liposomes without **IL**. Data are presented as mean ± standard deviation (*n* = 3).

**Figure 4:**
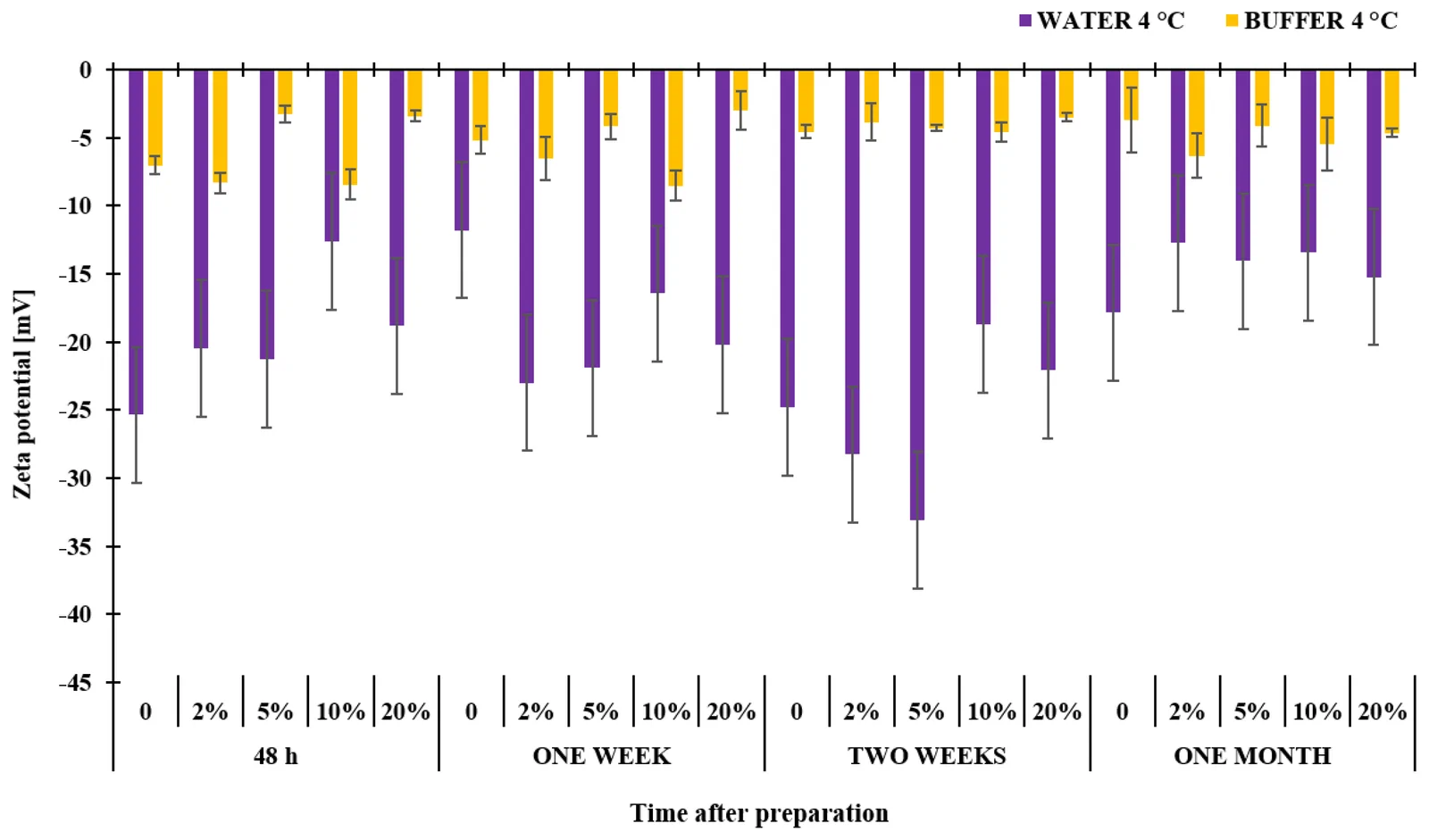
Zeta potential of DPPC/chol liposomes containing 0, 2, 5, 10, and 20 mol% **IL**, with a total lipid concentration of 0.13 mg/mL, measured after 48 h, one week, two weeks, and one month of storage. “0” means DPPC/chol liposomes without **IL**. Data are presented as mean ± standard deviation (*n* = 3).

The polydispersity index (PDI), hydrodynamic diameter, and zeta potential of the samples stored at RT were determined 2 h after preparation and after 48 h, one week, two weeks, and one month of storage. For samples stored at 4 °C, the same parameters were evaluated after 48 h, one week, two weeks, and one month of storage (Figure 2, Figure 3, Figure 4 and Figures S2, S12 and S13). Representative DLS particle size distribution profiles obtained for selected liposomal formulations are provided in the Supplementary Materials (Figures S3–S11) to provide additional information on the size distribution of the analyzed liposome populations.

The PDI was used to assess the homogeneity of the liposome population. Lower PDI values indicate a narrower particle size distribution, while higher values indicate greater heterogeneity and may reflect aggregation or other sources of particle-size variability. Low PDI values, close to zero, indicate a highly uniform particle size distribution, while values above 0.4 suggest a more heterogeneous and polydisperse particle population [30]. For lipid-based nanocarrier systems, including liposomes, PDI values of ≤0.3 are generally considered indicative of an acceptable degree of size homogeneity [31].

Formulations hydrated with milli-Q water and stored at 4 °C showed low and relatively stable PDI values throughout the observation period. As shown in Figure 2, the PDI values for the sample containing 20 mol% **IL** ranged from 0.04 to 0.21 during storage, remaining below the threshold of 0.3 generally considered acceptable for lipid-based nanocarriers. The control liposomes showed similarly low PDI values (0.03–0.12). The 10 mol% sample showed a similarly stable profile, with PDI values remaining between 0.04 and 0.12 during storage. The 2 mol% and 5 mol% samples also maintained low PDI values under these conditions. Thus, storage at 4 °C and hydration with milli-Q water favored the formation of a more homogeneous liposome population, even at the highest **IL** concentration tested.

Conversely, storage at RT resulted in a gradual increase in PDI for several formulations, particularly those hydrated with milli-Q water. The most pronounced changes occurred for the 20 mol% sample, for which the PDI increased from 0.19 after 48 h to 0.54 after one week and 0.82 after one month, exceeding 0.4, consistent with a highly heterogeneous particle population (Figure 2). PDI also increased for the 2, 5, and 10 mol% samples, although the magnitude of the change was lower than that observed for the 20 mol% **IL** formulation. The corresponding control achieved a PDI of 0.26 after one month.

Liposomes hydrated with PBS generally exhibited higher PDI values than their corresponding water-hydrated formulations (Figure S2). Under refrigerated conditions, the PDI of the 20 mol% sample remained in the range of 0.36–0.57 during storage. At RT, the 20 mol% **IL**-containing liposomes showed PDI values of 0.38–0.60. Overall, the lowest and most stable PDI values were observed for formulations hydrated with milli-Q water and stored at 4 °C.

The hydrodynamic diameter provides additional information on changes in liposome size and colloidal stability during storage. Changes in particle size can reflect processes such as aggregation or structural rearrangement and therefore provide useful information on the physical stability of liposome formulations. Therefore, maintaining a stable hydrodynamic diameter during storage can be considered an indicator of good colloidal stability of liposomes [32].

The most stable profiles were obtained for liposomes hydrated with milli-Q water and stored at 4 °C (Figure 3). Importantly, after one month, all **IL**-containing samples remained within a narrow range of approximately 141–145 nm, comparable to the control formulation (137 nm). Therefore, all **IL**-containing formulations maintained hydrodynamic diameters within a narrow nanoscale range under these storage conditions. The 10 mol% sample exhibited a particularly stable profile, for which the hydrodynamic diameter ranged from approximately 138 to 143 nm.

PBS-hydrated liposomes stored at 4 °C also fell within the nanoscale range, although particle size varied more substantially (Figure S12). The 10 mol% formulation exhibited hydrodynamic diameters of approximately 132–147 nm during storage, whereas the 20 mol% sample showed greater variability, with values ranging from approximately 168 to 205 nm. Therefore, compared with PBS, milli-Q water provided a more consistent particle size profile, particularly for the 20 mol% **IL** sample during refrigerated storage.

Several formulations stored at RT exhibited markedly different behavior, particularly those hydrated with milli-Q water. The most pronounced change occurred for the 20 mol% **IL** sample, for which the hydrodynamic diameter increased from 141 nm after 48 h to 241 nm after one week, 2130 nm after two weeks, and 2427 nm after one month (Figure S12). This marked increase, together with the corresponding increase in PDI, is consistent with progressive aggregation and loss of colloidal homogeneity. The 2 mol% sample also showed an increase, reaching a diameter of 409 nm after two weeks. Overall, the results indicate that storage temperature has a strong influence on the physical stability of the liposomal system.

PBS-hydrated liposomes stored at RT generally exhibited larger changes in particle size than their corresponding water-hydrated counterparts (Figure S12). After one month, the hydrodynamic diameters of the 2, 5, 10, and 20 mol% **IL** formulations were within approximately 156–201 nm. However, the most consistent particle size profiles were observed for the milli-Q water-hydrated formulations stored at 4 °C.

The final parameter evaluated as an indicator of liposome stability was the zeta potential, which provides information about the electrostatic properties of liposome surfaces and can therefore be used to assess the tendency of vesicles to remain dispersed in suspension. A sufficiently high absolute zeta potential can increase the electrostatic repulsion between adjacent vesicles and thereby reduce their tendency to approach each other and aggregate [31,33].

Zeta potential measurements showed a clear dependence on the hydration medium (Figure 4 and Figure S13). Water-hydrated liposomes generally exhibited more negative zeta potential values than their PBS-hydrated counterparts. This difference is consistent with the effect of ionic strength on the measured surface charge of colloidal particles, as ions present in the buffer can partially screen the surface charge [34,35,36,37].

Milli-Q water-hydrated liposomes stored at 4 °C maintained negative zeta potential values throughout the observation period. Specifically, the 20 mol% **IL** sample exhibited zeta potential values of approximately −19 to −22 mV during most of the observation period, with a less negative value of −15 mV after one month (Figure 4). The 10 mol% **IL** sample exhibited a comparable profile, with values of approximately −13 to −19 mV. Therefore, despite the highest concentration of **IL** tested, the 20 mol% formulation retained a negative surface potential with only limited changes during storage at 4 °C. The limited changes in zeta potential, along with the stable hydrodynamic diameter and low PDI observed under these conditions, further support the preservation of the colloidal state of this formulation. The zeta potential of the 20 mol% **IL** formulation remained within a range comparable to that observed for the corresponding control formulation, although the **IL**-containing sample generally exhibited less negative values at some measurement times.

In contrast, the 20 mol% sample hydrated with milli-Q water and stored at RT exhibited greater zeta potential variability during long-term storage (Figure 4). This variability coincided with a marked increase in hydrodynamic diameter and PDI measured for the same formulation, suggesting that the surface charge changes were part of a broader colloidal destabilization process.

PBS-hydrated formulations exhibited less negative zeta potential values, generally ranging from approximately −12 to −3 mV (Figure S13). PBS buffer was employed primarily to assess the colloidal stability of the liposomes under physiologically relevant conditions. However, due to significant electrostatic charge screening at high ionic strength, zeta potential values in PBS collapsed to near-neutral values (−5 mV), precluding definitive conclusions regarding **IL**-induced surface charge modifications in buffered media.

In summary, the DLS and ELS results demonstrate that both the hydration medium and storage temperature influence the physicochemical stability of **IL**-containing liposomes. The most favorable stability profile was observed for liposomes hydrated with milli-Q water and stored at 4 °C, as reflected by the maintenance of low PDI values, a relatively stable hydrodynamic diameter, and a negative zeta potential throughout the storage period. These findings are consistent with previous reports on the stability and long-term integrity of DPPC/cholesterol-based liposomes. DPPC-based liposomal systems containing cholesterol have been shown to exhibit high structural stability, supporting their storage over extended periods beyond one month, as demonstrated, for example, by commercially available clodronate-loaded liposomes [38]. Furthermore, Xu et al. reported that, following formulation optimization, liposomes in aqueous dispersion maintained stable particle size and membrane integrity for at least 6 months at 4 °C [39].

Under the conditions tested in the present study, both the 10 and 20 mol% **IL**-containing liposomes maintained satisfactory colloidal stability during storage at 4 °C following hydration with milli-Q water. Although the 10 mol% **IL** formulation showed somewhat more stable PDI values and, under some storage conditions, better colloidal stability than the 20 mol% formulation, both formulations retained satisfactory physicochemical properties under the selected storage conditions. The 20 mol% **IL** formulation was therefore selected for further characterization as it provided the highest **IL** content while maintaining satisfactory colloidal stability under the selected storage conditions. Thus, the selection of the 20 mol% **IL** formulation represented a trade-off between **IL** content and colloidal stability rather than indicating superior overall stability compared with the 10 mol% formulation. This formulation was subsequently used to investigate the effect of **IL** incorporation on DPPC/chol bilayer organization and biophysical properties using fluorescence and infrared spectroscopy, as well as to determine the **IL** loading efficiency of the liposomes.

### 2.2. Determination of the Loading Efficiency of Liposomes with **IL**

Loading efficiency (LE%) is an important parameter in the characterization of liposomal formulations, as it provides information on the fraction of the compound associated with the liposomal carrier relative to the total amount initially used for the formulation process [40].

Loading efficiency was defined as the proportion of **IL** retained within the liposomal fraction relative to the initial amount of **IL**. Quantification was achieved by separating the free **IL** via ultracentrifugation, followed by liquid–liquid extraction and gas chromatography (GC) analysis. Control DPPC/chol liposomes and liposomes loaded with 20 mol% **IL** were evaluated. Both systems were hydrated with milli-Q water, and LE% was determined immediately after liposome formation.

Quantitative analysis of **IL** by GC was based on a calibration curve prepared using **IL** standard solutions in the concentration range of 0–2 mg/mL. The calibration curve demonstrated good linearity over the entire concentration range tested (R^2^ = 0.9949, Figure S14 in Supplementary Materials). No signals corresponding to **IL** were detected in the control liposome sample.

The LE% was determined based on the difference between the initial amount of **IL** used for liposome formulation and the amount of **IL** detected in the aqueous supernatant after ultracentrifugation and subsequent extraction. The 20 mol% **IL** formulation showed an LE% of 87.6 ± 7.8% (mean ± SD; *n* = 3). The corresponding amount of **IL** associated with the liposomal fraction was 0.127 mg, compared with 0.145 mg initially used for liposome preparation. This indicates that most of the **IL** initially used for the 20 mol% formulation (0.145 mg) was loaded into the DPPC/chol liposomal fraction. The obtained results demonstrate the suitability of the developed methodology for quantifying the **IL** fraction not loaded into the liposomes, thereby enabling the determination of LE%.

The recovery efficiency of the applied procedure was additionally assessed using a recovery experiment, in which 0.145 mg of **IL** was subjected to the same extraction/analysis procedure, and 0.134 mg was recovered and quantified by GC, corresponding to a recovery of 92.83%. This high recovery rate falls well within acceptable analytical limits, validating the procedure for accurate indirect quantification of loading efficiency. A limitation of this procedure is that partial sedimentation of free, unloaded **IL** during ultracentrifugation cannot be completely ruled out. In this case, a fraction of the sedimented **IL** would not be associated with the liposomes. Therefore, the reported value LE% serves as an indirect estimate of liposomal loading efficiency.

### 2.3. Effect of **IL** on Liposomal Bilayer Properties

#### 2.3.1. Fluorescence Probe-Based Assessment of Liposomal Bilayers

**Figure 5:**
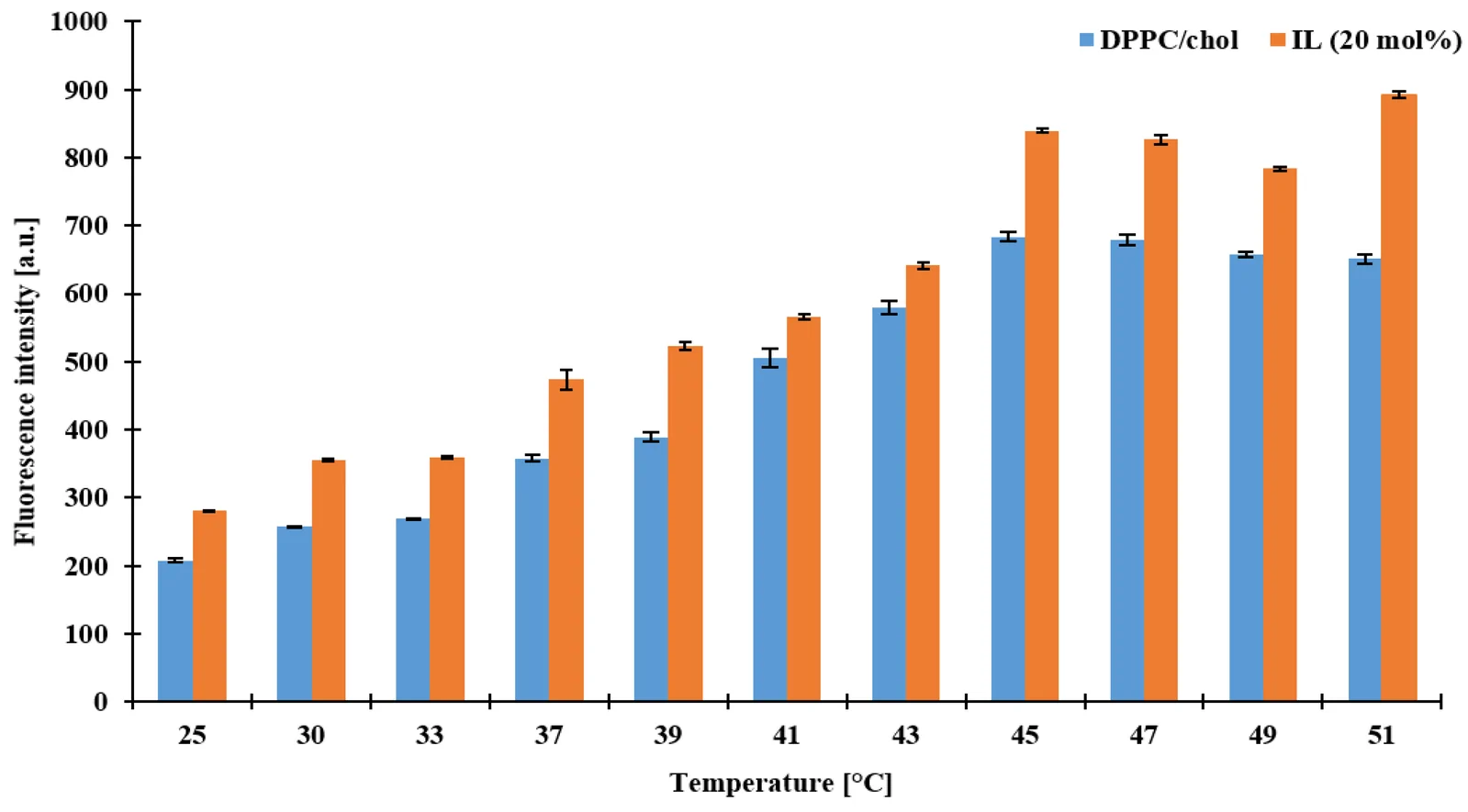
Temperature-dependent changes in MC540 fluorescence intensity in liposomes and liposomes containing 20 mol% **IL**, with a total lipid concentration of 0.13 mg/mL, at λ = 587 nm. “DPPC/chol” means DPPC/chol liposomes without **IL**. Data are presented as mean ± standard deviation (*n* = 3). Statistical analysis (Figure S16 in Supplementary Materials) was performed using two-way analysis of variance (ANOVA), followed by Šídák’s multiple comparisons test for multiple comparisons between groups.

**Figure 6:**
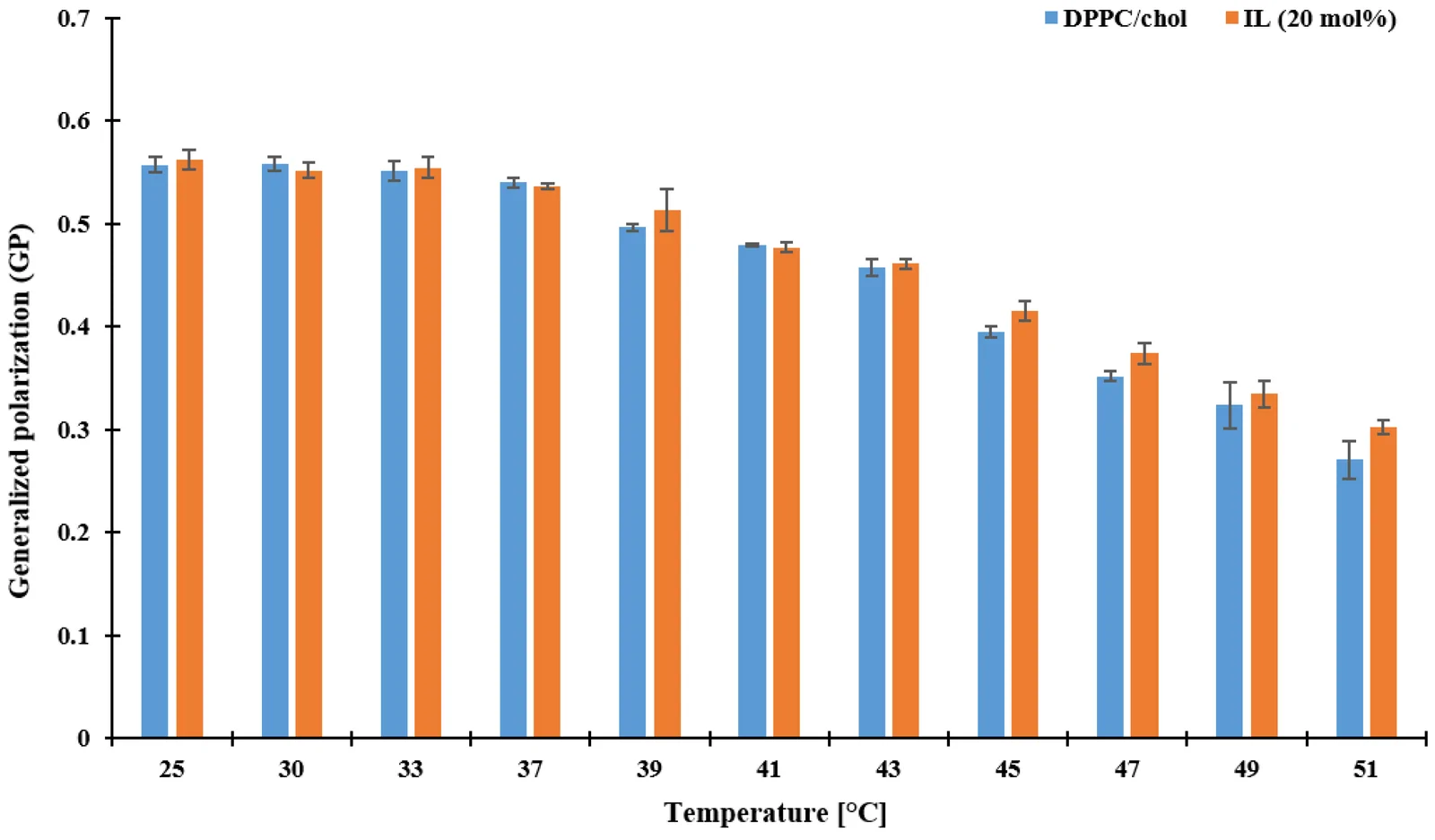
Temperature-dependent changes in Laurdan generalized polarization (GP) in control liposomes and liposomes containing 20 mol% **IL**, with a total lipid concentration of 0.13 mg/mL. “DPPC/chol” means DPPC/cholesterol liposomes without **IL**. Data are presented as mean ± standard deviation (*n* = 3). Statistical analysis (Figure S17 in Supplementary Materials) was performed using two-way analysis of variance (ANOVA), followed by Šídák’s multiple comparisons test for multiple comparisons between groups.

**Figure 7:**
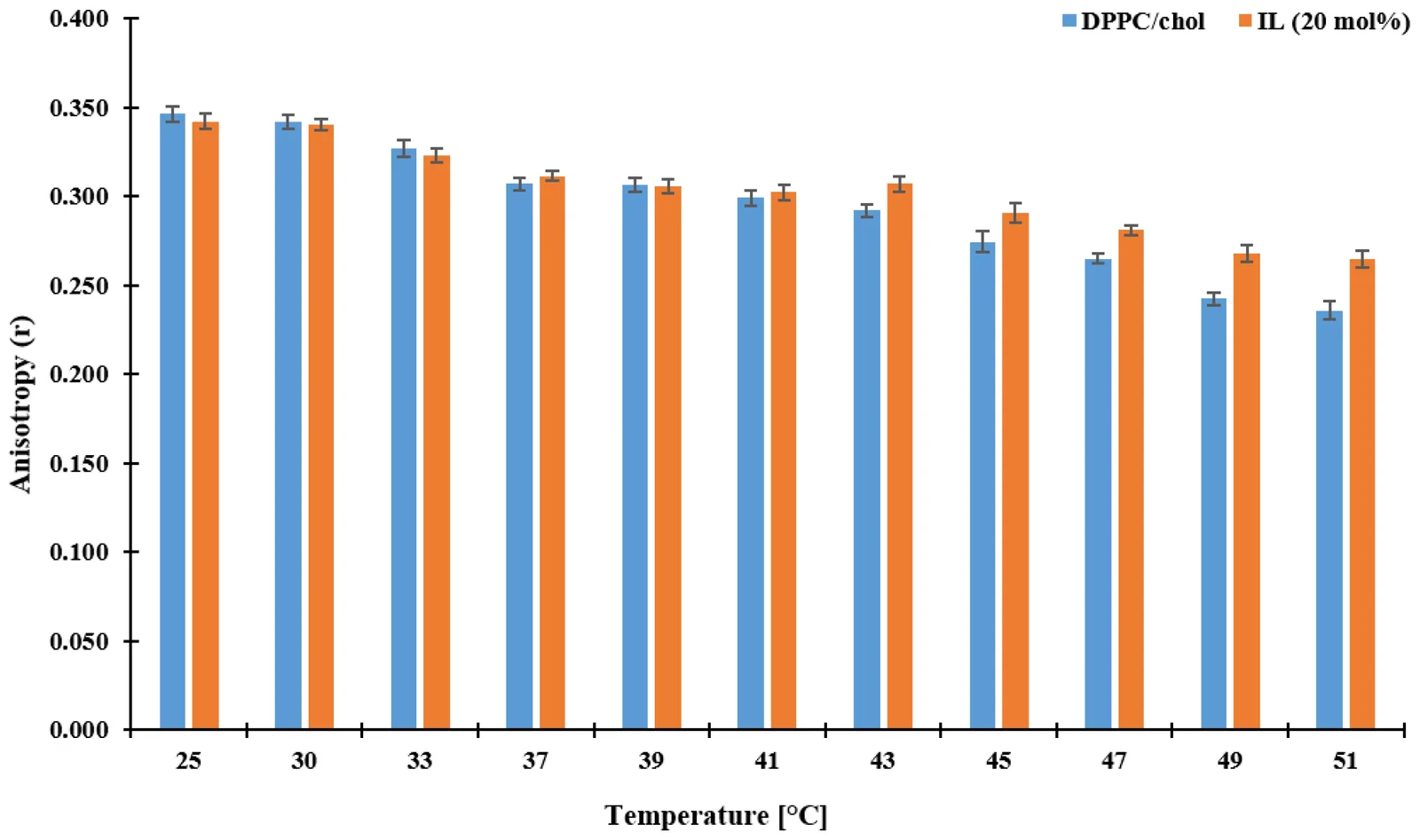
Temperature-dependent changes in DPH fluorescence anisotropy (r) in control liposomes and liposomes containing 20 mol% **IL**, with a total lipid concentration of 0.13 mg/mL. “DPPC/chol” means DPPC/cholesterol liposomes without **IL**. Data are presented as mean ± standard deviation (*n* = 3). Statistical analysis (Figure S18 in Supplementary Materials) was performed using two-way analysis of variance (ANOVA), followed by Šídák’s multiple comparisons test for multiple comparisons between groups.

To evaluate the effect of **IL** at the highest concentration (20 mol%) on the physicochemical properties of the liposomal membrane, fluorescence spectroscopy measurements were performed using three membrane probes: merocyanine 540 (MC540), 1-[6-(dimethylamino)naphthalen-2-yl]dodecan-1-one (Laurdan), and 1,6-diphenyl-1,3,5-hexatriene (DPH). The chemical structures of the fluorescent probes are provided in the Supplementary Materials (Figure S15). The probes were used to characterize different regions and properties of the lipid bilayer, including lipid packing at the membrane interface, membrane hydration and interfacial lipid order (MC540 and Laurdan), and the dynamic properties of the hydrophobic acyl chain region (DPH) (Figure 5, Figure 6 and Figure 7).

MC540 preferentially associates with loosely packed lipid domains, and its fluorescence properties are sensitive to the local membrane environment, including lipid packing, probe orientation, and partitioning between aqueous and hydrophobic regions [41,42,43]. An increase in MC540 fluorescence intensity indicates decreased packing and increased accessibility of the outer layer of the liposomal membrane to the probe, while a decrease in fluorescence intensity suggests denser lipid packing and reduced accessibility of the probe [44].

The temperature-dependent fluorescence intensity of MC540 in DPPC/chol liposomes and liposomes containing 20 mol% **IL** is shown in Figure 5. In the control samples, MC540 fluorescence gradually increased with temperature from 208.1 ± 2.4 at 25 °C to 683.4 ± 7.1 at 45 °C and then decreased to 650.9 ± 6.7 at 51 °C. In comparison, the **IL**-containing liposomes showed consistently higher fluorescence intensity at all temperatures tested, reaching 281.2 ± 1.0 at 25 °C, 473.9 ± 14.8 at 37 °C, 839.7 ± 3.1 at 45 °C, and 892.7 ± 4.3 at 51 °C (Figure 5). Thus, the presence of **IL** increased MC540 fluorescence compared with the control by approximately 35% at 25 °C, 32% at 37 °C, 23% at 45 °C, and 37% at 51 °C. Statistical analysis (Figure S16 in the Supplementary Materials) confirmed that the fluorescence intensity was significantly higher in **IL**-containing liposomes than in control liposomes at every temperature tested (25–51 °C; Šídák-adjusted *p* < 0.0001 for all comparisons). These observations suggest that the incorporation of **IL** may alter the organization and surface properties of the lipid bilayer, potentially affecting probe accessibility and partitioning. However, potential contributions from direct interactions between **IL** and MC540—such as altered probe partitioning, aggregation state, or intrinsic optical properties—cannot be completely excluded and represent a plausible additional factor contributing to the increased signal.

To extend the investigation of the effects of **IL** on lipid bilayer organization, a study was conducted using the Laurdan probe. The results obtained using the Laurdan probe were expressed as generalized polarization (GP) values, which provide information on the polarity and hydration of the probe’s local membrane environment and are widely used to characterize lipid ordering and membrane phase state [45,46,47]. Higher GP values generally indicate a more ordered, tightly packed, and less hydrated membrane environment, whereas lower GP values are associated with increased water penetration, greater membrane fluidity, and reduced lipid ordering [48].

The GP values obtained at different temperatures are presented in Figure 6. In both tested formulations, GP gradually decreased with increasing temperature, probably indicating increased hydration of the local probe environment within the lipid bilayer and a concomitant decrease in lipid ordering. In control liposomes, GP decreased from 0.557 ± 0.007 at 25 °C to 0.395 ± 0.006 at 45 °C and further to 0.271 ± 0.019 at 51 °C. The corresponding values for the **IL**-containing sample were 0.562 ± 0.009, 0.415 ± 0.009, and 0.302 ± 0.007, respectively (Figure 6). Thus, the **IL**-containing formulation exhibited higher GP values than the control at most temperatures, and this difference became more pronounced with increasing temperature. At 45 °C, GP was approximately 5% higher in the **IL**-containing formulation than in the control, whereas at 51 °C the difference increased to approximately 12%. By contrast, at 25 °C, both formulations exhibited very similar GP values (0.557 versus 0.562). However, statistical analysis (Figure S17 in the Supplementary Materials) showed that differences were not significant in the temperature range of 25–49 °C (*p* > 0.05). A statistically significant difference between the formulations was observed only at 51 °C (Šídák-adjusted *p* < 0.01), where GP was 0.0317 higher in the **IL**-containing liposomes than in the control. These results indicate that **IL** significantly affected membrane organization only at the highest temperature tested, whereas no statistically significant effect was observed at the other temperatures. At 51 °C, the higher GP value in the **IL**-containing liposomes may reflect a lower degree of membrane hydration and/or greater interfacial ordering compared with the control.

The effect of **IL** on the hydrophobic region of the lipid bilayer was further investigated by measuring DPH fluorescence anisotropy (r). This probe is incorporated into the hydrophobic core of lipid bilayers, and the resulting fluorescence anisotropy provides information on the rotational mobility and acyl chain ordering [49,50]. Higher anisotropy values indicate more restricted probe rotation and, consequently, greater lipid ordering in the hydrophobic region [51].

The raw DPH excitation spectra and polarized fluorescence intensity components used for the analysis are provided in the Supplementary Materials (Figures S19 and S20).

The obtained anisotropy values at different temperatures are shown in Figure 7. In the control sample, the anisotropy gradually decreased from 0.346 ± 0.005 at 25 °C to 0.307 ± 0.004 at 37 °C and 0.236 ± 0.005 at 51 °C, indicating increased mobility of the acyl chains in the hydrophobic region with increasing temperature. **IL**-containing liposomes exhibited very similar anisotropy at 25 °C (0.342 ± 0.004), indicating little difference compared with the control at the lowest temperature tested. At 37 °C, the anisotropy was 0.311 ± 0.003 in the tested compound-containing formulation compared to 0.307 ± 0.004 in the control, whereas at 45 °C the corresponding values were 0.290 ± 0.005 and 0.274 ± 0.006, respectively. The largest difference occurred at 51 °C, where the anisotropy of the **IL**-containing sample reached 0.265 ± 0.005 compared to 0.236 ± 0.005 in the control (Figure 7). This corresponds to approximately 12% higher anisotropy in the presence of **IL** at 51 °C. Thus, both formulations exhibited increased acyl chain mobility upon heating, while the differences in anisotropy between the formulations remained relatively small. Statistical analysis (Figure S18 in the Supplementary Materials) showed that anisotropy differed significantly between the formulations at 30, 33, 43, 45, 47, 49, and 51 °C (Šídák-adjusted *p* < 0.0001), whereas no significant differences were observed at 25, 37, 39, or 41 °C (Šídák-adjusted *p* > 0.05). At temperatures above 43 °C, the **IL**-containing liposomes showed slightly higher anisotropy than the control, which may indicate a modest reduction in temperature-induced acyl chain mobility. However, the absence of a statistically significant difference at 37 °C, together with the relatively small absolute differences in anisotropy, suggests that the effect of **IL** on acyl chain mobility was limited under physiologically relevant temperature conditions.

In summary, fluorescence spectroscopy results obtained with three complementary probes indicate that **IL** modifies the temperature-dependent organization of the DPPC/chol bilayer compared with the control. MC540 measurements showed consistently higher fluorescence in the **IL**-containing formulation, with statistically significant differences observed at all temperatures tested, suggesting changes in membrane packing and/or probe accessibility. However, contributions from altered MC540 partitioning, aggregation state, or optical properties in the presence of **IL** cannot be excluded. Laurdan measurements showed a general tendency toward higher GP values in the **IL**-containing formulation, particularly at elevated temperatures, although the difference between formulations reached statistical significance only at 51 °C, with the higher GP at 51 °C potentially indicating a lower degree of membrane hydration and/or greater interfacial ordering under this condition. A similar temperature-dependent difference was observed for DPH, with the **IL**-containing formulation showing slightly higher anisotropy than the control at elevated temperatures, although the absolute differences in anisotropy were relatively small. No statistically significant difference was observed at 37 °C, whereas significant differences were observed at several temperatures above 43 °C. At these higher temperatures, the slightly higher anisotropy may indicate a modest reduction in temperature-induced acyl chain mobility in the presence of **IL**. Thus, the magnitude and statistical significance of the effects varied depending on the fluorescent probe and temperature. The differences between the **IL**-containing and control liposomes were most evident at elevated temperatures, whereas no statistically significant differences were observed at 37 °C for either Laurdan or DPH.

The lack of a sharp transition observed in this study suggests that the DPPC/chol bilayer maintains a relatively stable organization over the tested temperature range, although temperature-dependent changes in membrane organization were still observed. Cholesterol is known to strongly influence membrane order and phase behavior and can alter the thermal behavior of phospholipid bilayers [52]. At a concentration of 20 mol% cholesterol in the DPPC bilayer, characteristic changes occur in the thermotropic behavior and membrane structure. The main phase transition of DPPC becomes markedly broadened and non-cooperative. At temperatures above this transition, cholesterol increases the conformational ordering of the aliphatic acyl chains of DPPC and induces the formation of a unique liquid-ordered phase. Conversely, at low temperatures (below the main phase transition), it introduces conformational disorder and increases the mobility of the polar lipid headgroups [53].

The findings of the present study are consistent with those previously reported for **IL** in three different model membranes: membranes based on erythrocyte lipid extracts, erythrocyte membranes, and lipid membranes mimicking tumor cells. Measurements of **IL** using MC540, Laurdan, and DPH also indicated moderate but concentration-dependent changes in membrane organization, with more pronounced effects at the membrane interface than in the hydrophobic core [26]. The results obtained in this study can also be compared with those reported by Gładkowski et al., who investigated chiral *trans*-δ-bromo-γ-lactones derived from 2,5-dimethylbenzaldehyde in erythrocyte membranes using the same three fluorescent probes at 37 °C [54]. The tested compounds showed a minor effect on the hydrophobic region, as indicated by DPH anisotropy values remaining at the control level, whereas changes detected by MC540 and Laurdan were observed mainly in the interfacial region of the membrane. A similar pattern was observed in the present DPPC/chol system, where **IL** affected both the interfacial and hydrophobic regions of the membrane.

#### 2.3.2. ATR-FTIR Analysis of Liposomal Bilayers

ATR-FTIR spectral analysis of the DPPC/chol bilayer in the presence of **IL** enabled the precise determination of its molecular localization and its impact on the structural organization of lipids, based on the frequency shifts and profile alterations of diagnostic bands. The infrared absorption spectrum of the DPPC/chol model system is characterized by bands originating from both the polar and nonpolar regions of the bilayer, as well as from the steroid skeleton of cholesterol (Figure S21 in Supplementary Materials). In the high-wavenumber region (2800–3000 cm^−1^), high-intensity bands dominate, corresponding to the C-H stretching vibrations of the DPPC acyl chains and cholesterol molecules (Figure S21). The maximum at 2918 cm^−1^ was assigned to the asymmetric stretching vibrations of methylene groups (ν_as_(CH_2_)), whereas the band at 2850 cm^−1^ corresponds to their symmetric stretching vibrations (ν_s_(CH_2_)). The presence of a prominent signal at 2956 cm^−1^ is associated with the asymmetric stretching vibrations of methyl groups ν_as_(CH_3_) originating from the terminal methyls of the palmitic chains and the methyl groups of cholesterol (Table 1).

In the mid-wavenumber region, deformation vibration bands of aliphatic groups were recorded: methylene scissoring vibrations (δ(CH_2_)) appearing around 1467 cm^−1^ and symmetric methyl bending vibrations (δ_s_(CH_3_)) centered at approximately 1377 cm^−1^. The signal centered at approximately 721 cm^−1^ corresponds to the rocking vibrations (γ_r_(CH_2_)) of methylene sequences in the all*-trans* conformation. In the interfacial region, a distinct band at 1737 cm^−1^ is observed, attributed to the carbonyl stretching vibrations ν(C=O) of the ester bonds linking the palmitic acid residues to the glycerol backbone. The C-O stretching vibrations of the ester linkage in the *sn*-2 position appear centered at approximately 1171 cm^−1^ (ν_as_(COC)). The polar region of the DPPC/chol bilayer is represented by two intense phosphate group bands: asymmetric stretching vibrations ($\nu_{as}\left({\mathrm{PO}}_{2}^{-}\right)$) at 1232–1243 cm^−1^ and symmetric stretching vibrations ($\nu_{s}\left({\mathrm{PO}}_{2}^{-}\right)$) centered at approximately 1089 cm^−1^. Vibrations of the phosphodiester backbone (ν(C-O-P)) are observed at 1066 cm^−1^. Meanwhile, the band appearing around 970 cm^−1^ was assigned to the asymmetric N-C stretching vibrations (ν_as_(N-C)) of the quaternary trimethylammonium group in the choline headgroup [55] (Table 1).

Incorporation of the vanillin-derived lactone into the DPPC/cholesterol bilayer leads to subtle spectral perturbations in the lipid matrix bands [56,57]. The C-H stretching vibration bands ν(CH_2_) serve as a particularly sensitive indicator of the conformational order and fluidity of the aliphatic fatty acid chains within the bilayer. The absence of significant shifts in the absorption maxima for both symmetric (ν_s_(CH_2_)) and asymmetric (ν_as_(CH_2_)) vibrations indicates the lack of structural perturbations within the hydrocarbon chains (Table 1). The preservation of constant wavenumber values in the presence of **IL** demonstrates that the compound neither induces defects within the hydrophobic region nor destabilizes acyl chain packing. Furthermore, the symmetric and asymmetric stretching bands exhibit negligible variation across the entire temperature range (25–51 °C). The absence of a stepwise frequency increase in the ν(CH_2_) bands confirms that the bilayer remains in the cholesterol-stabilized liquid-ordered phase [53].

The observed alterations in the profile and symmetry of the lipid ester carbonyl stretching band (ν(C=O)) centered at approximately 1737 cm^−1^ reflect subtle molecular perturbations within the interfacial region of the bilayer upon compound incorporation (Figure S21). As reported in our previous work [25], the pure vanillin-derived lactone exhibits a characteristic, diagnostic γ-lactone C=O stretching band at 1770.91 cm^−1^ due to ring strain. In the liposomal formulations, however, no isolated peak at 1770 cm^−1^ is observed; instead, the lactone signal merges directly into the broad lipid ester envelope. This absence of a discrete compound peak confirms that the lactone is intercalated within the lipid matrix rather than forming a separate crystalline phase. Because direct peak-picking of the broad ester envelope reveals only minor frequency variations, Gaussian curve-fitting deconvolution was applied to resolve the underlying populations (Figure S22). This analysis demonstrates that compound incorporation shifts the equilibrium toward the high-frequency non-hydrated component (1742 cm^−1^) at the expense of the hydrated population (1733 cm^−1^), indicating partial dehydration of the glycerol backbone driven by water displacement upon heterocyclic ring insertion. These localized perturbations within the ν(C=O) region, together with subtle shifts in the ν_as_(C-O-C)_sn2_ stretching band from 1171.02 ± 0.07 cm^−1^ to 1172.08 ± 0.13 cm^−1^ (Table 1), suggest the effective anchoring of the lactone ring near the glycerol backbone level [58].

The asymmetric stretching band of the phosphate groups ($\nu_{as}\left({\mathrm{PO}}_{2}^{-}\right)$) shifts toward higher wavenumbers with increasing temperature, from 1231.97 ± 0.64 cm^−1^ at 25 °C to 1243.15 ± 0.07 cm^−1^ at 51 °C, due to the reorganization of the hydration shell (Table 1). Unlike the pure DPPC bilayer, which undergoes a gel-to-liquid crystalline phase transition accompanied by a sudden expansion of headgroup surface area and increased hydration, the inclusion of 20 mol% cholesterol establishes a liquid-ordered phase. In this state, the sharp phase transition is abolished, and elevating the temperature predominantly promotes thermally induced dehydration, as higher thermal kinetic energy disrupts weak hydrogen bonds between water molecules and phosphate oxygens. Across all analysed temperatures, the presence of the lactone downshifts the $\nu_{as}\left({\mathrm{PO}}_{2}^{-}\right)$ band frequency by 0.8–1.2 cm^−1^ (e.g., from 1241.65 ± 0.82 cm^−1^ to 1240.47 ± 0.74 cm^−1^ at 37 °C), suggesting the formation of hydrogen bonds or electrostatic interactions with the DPPC phosphate groups [55,59]. A slight downshift in the frequencies of the choline headgroup bands, ν_as_(N-C) and ν_s_(N-C) (Table 1), further reflects modifications to the polar environment at the very surface of the membrane. Conversely, the negligible alterations in the δ(CH_2_) vibrations (≤0.2 cm^−1^) indicate the preservation of packing stability within the hydrophobic region.

The absence of pronounced shifts in the acyl chain stretching vibrations (ν(CH_2_)) when cross-referenced with spectrofluorometric data suggests that membrane reorganization occurs predominantly within the polar and interfacial regions, with limited penetration into the hydrophobic core or disruption of bilayer continuity. The downshift observed in the asymmetric phosphate stretching frequency ($\nu_{as}\left({\mathrm{PO}}_{2}^{-}\right)$) across all measured temperatures, together with subtle shifts in the choline bands (ν_as_(N-C^+^)), is consistent with increased hydrogen bonding or electrostatic interactions at the membrane surface. This localized alteration of headgroup packing likely facilitates the partitioning of the MC540 probe into the subsurface zone, promoting its transition from a dimer to a more strongly fluorescent monomer. Furthermore, deconvolution of the ester carbonyl (C=O) band revealed a redistribution between the non-hydrogen-bonded (1742 cm^−1^) and hydrogen-bonded (1733 cm^−1^) components, indicating a localized structural rearrangement within the glycerol backbone region. While ATR-FTIR measurements conducted on dehydrated liposomes describe interfacial organization in the dehydrated state rather than direct hydration dynamics in bulk solution, these structural adjustments complementarily support the restricted dipolar relaxation detected by Laurdan GP in aqueous dispersion.

In summary, based on ATR-FTIR spectral analysis alongside complementary biophysical assays, these findings indicate that the **IL** is localized primarily within the interfacial region and the polar headgroup zone of the bilayer, residing at the boundary between the aqueous phase and the hydrophobic lipid core. DLS/ELS measurements further showed that **IL**-containing liposomes maintained favorable colloidal properties during storage at 4 °C after hydration with milli-Q water. Together with the spectroscopic data, these results support the view that **IL** modulates membrane organization predominantly at the polar surface, while exerting a relatively limited effect on the hydrophobic core.

### 2.4. Hemolytic Activity of **IL**-Loaded Crude Dispersion Fractions (CDFs) and Ultracentrifuged Liposomal Fraction (ULF)

**Figure 8:**
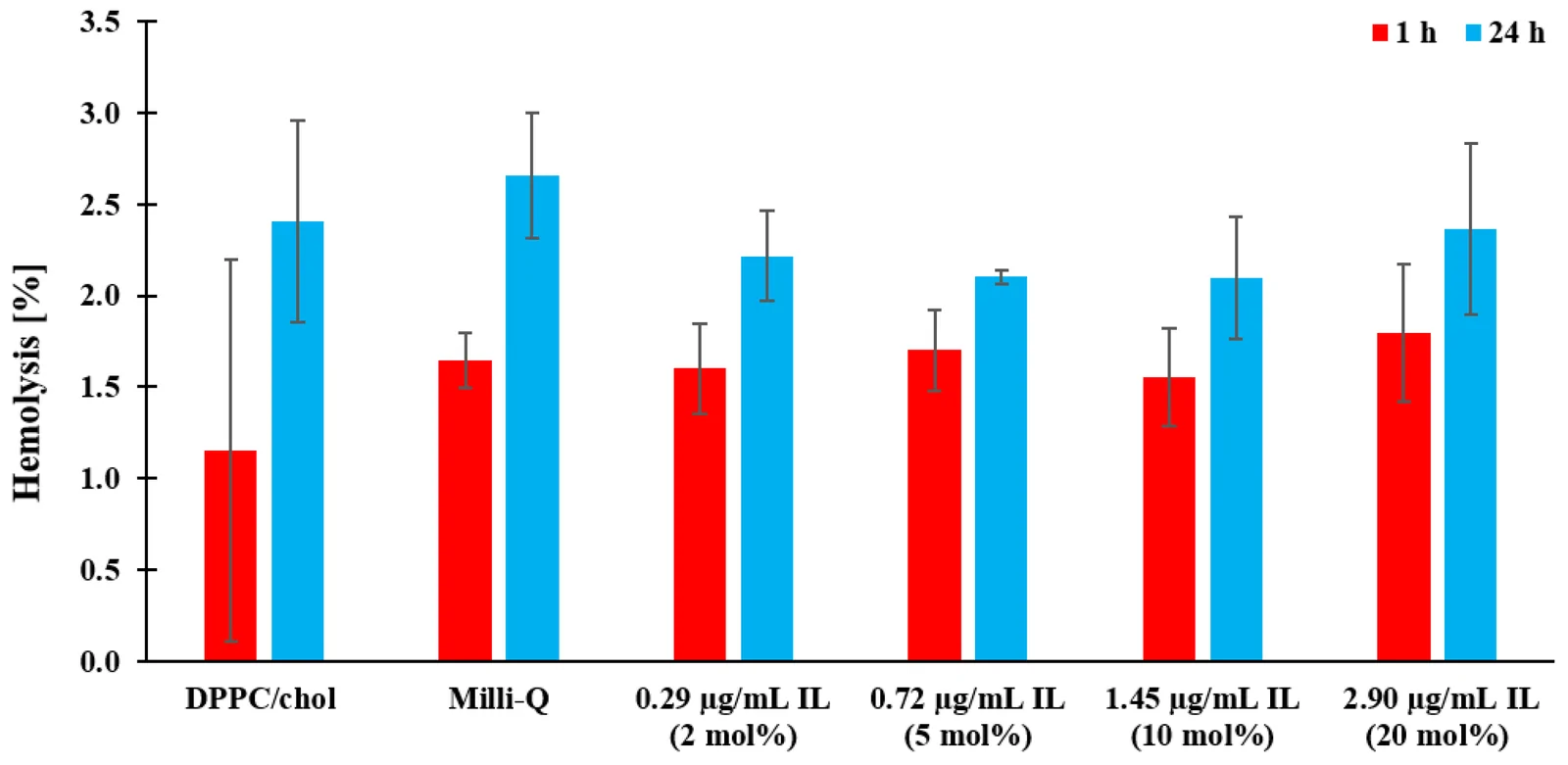
Hemolytic activity of CDFs containing 2, 5, 10, and 20 mol% **IL** at a total lipid concentration of 0.0266 mg/mL, after 1 and 24 h of incubation with RBCs at 37 °C. “DPPC/chol” refers to DPPC/cholesterol liposomes without **IL**, whereas “Milli-Q” refers to the negative control, in which **IL** was replaced with an equivalent volume of milli-Q water. Data are presented as mean ± SD (*n* = 3). Statistical analysis was performed using two-way analysis of variance (ANOVA), followed by Dunnett’s multiple comparisons test for comparisons with the control group.

**Figure 9:**
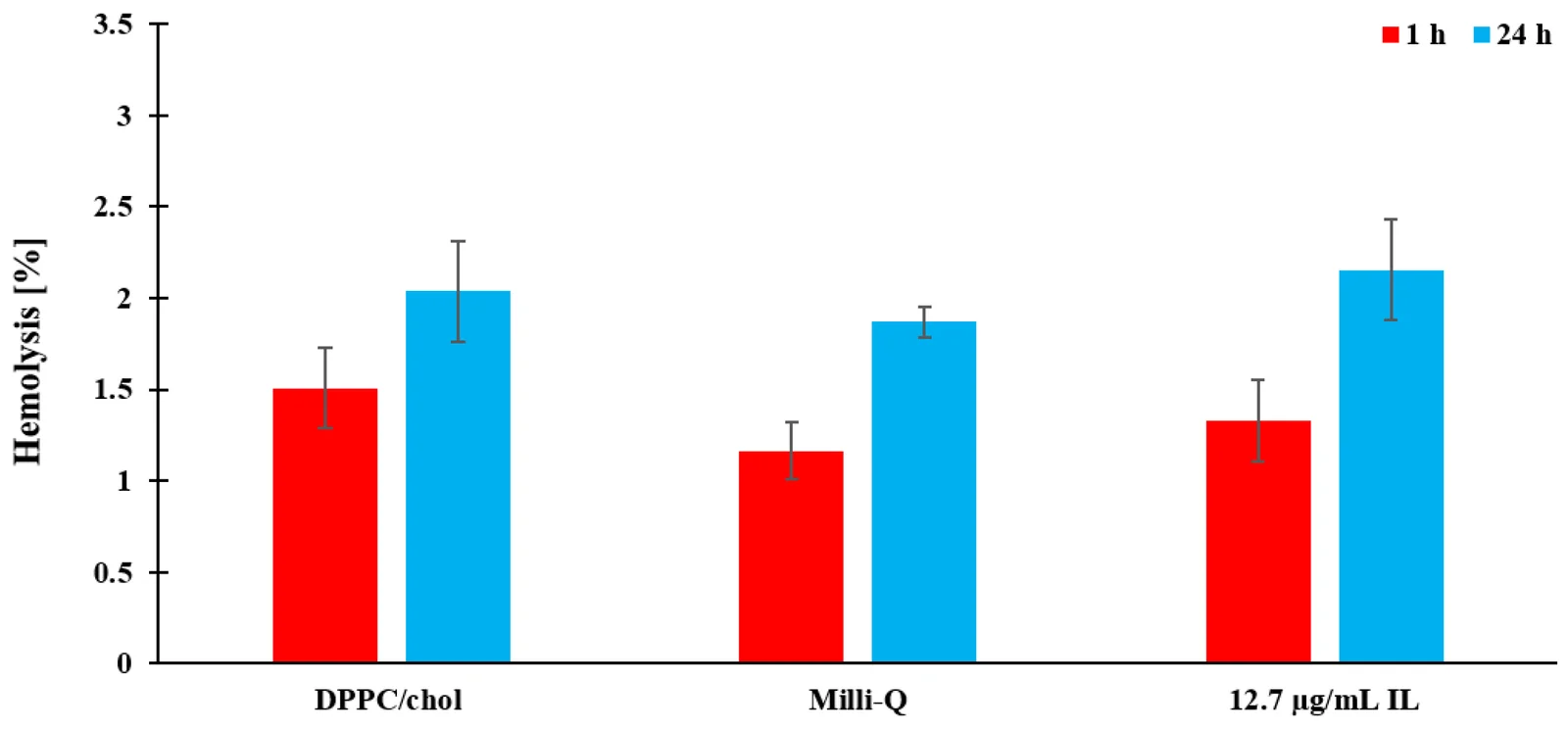
Hemolytic activity of ULF 20 mol% **IL**-loaded liposomes determined after 1 and 24 h of incubation with RBCs at 37 °C. “DPPC/chol” refers to DPPC/cholesterol liposomes without **IL**, whereas “Milli-Q” refers to the negative control, in which **IL** was replaced with an equivalent volume of milli-Q water. Data are presented as mean ± SD (*n* = 3). Statistical analysis was performed using two-way analysis of variance (ANOVA), followed by Dunnett’s multiple comparisons test for comparisons with the control group.

The hemolytic activity of **IL**-containing liposomal formulations was assessed using human red blood cells (RBCs) to evaluate the potential effects of the formulations on erythrocyte membranes. Hemolysis was determined after 1 and 24 h of incubation at 37 °C and expressed as a percentage relative to the positive control. Crude dispersion formulations (CDFs) containing increasing concentrations of **IL** (2, 5, 10, and 20 mol%) were tested (Figure 8 and Figure 9). The hemolytic activity of the formulations was assessed in comparison with the liposome control without **IL** and a milli-Q water control, allowing the effect of **IL** incorporation on erythrocyte membrane integrity to be evaluated.

For the CDFs, only low hemolytic activity was observed at any of the **IL** concentrations tested (Figure 8). After 1 h of incubation, the DPPC/cholesterol control liposomes induced hemolysis of 1.2 ± 1.0%, whereas formulations containing 2, 5, 10, and 20 mol% **IL** induced hemolysis of 1.6 ± 0.2%, 1.7 ± 0.2%, 1.6 ± 0.3%, and 1.8 ± 0.4%, respectively. These values were comparable to those for the negative control and the liposome control, indicating that the presence of **IL** did not result in a concentration-dependent increase in hemolysis after 1 h of incubation. In agreement with these observations, statistical analysis (Figure S23, Supplementary Materials) showed no statistically significant effect of **IL** concentration on hemolysis (*p* = 0.6446) and no significant interaction between **IL** concentration and incubation time (*p* = 0.5254). After 24 h, hemolysis remained low, ranging from 2.1 ± 0.0% to 2.4 ± 0.6% for the **IL**-containing formulations, compared with 2.4 ± 0.6% for the liposome control and 2.7 ± 0.3% for the milli-Q control (Figure 8). Although a significant overall effect of incubation time was observed (*p* < 0.0001; Figure S23, Supplementary Materials), indicating higher hemolysis after 24 h compared with 1 h, the lack of a significant interaction between incubation time and **IL** concentration indicates that the observed time-dependent increase in hemolysis was not dependent on **IL** concentration. Thus, even at the highest **IL** concentration tested (20 mol%), hemolysis remained low, comparable to that observed for the corresponding control formulations.

To better identify the effect of **IL** associated with the liposomal fraction from the effect of non-liposomal **IL** present in the aqueous phase of the formulation, the hemolytic activity of the **IL**-containing liposomal fraction after ultracentrifugation (ULF) was additionally assessed after removing the aqueous supernatant containing unbound **IL**.

As shown in Figure 9, the ULF containing **IL** showed similarly low hemolytic activity. After 1 h of incubation, hemolysis was 1.33 ± 0.22%, compared with 1.51 ± 0.22% for the liposome control and 1.16 ± 0.15% for the milli-Q control. After 24 h, hemolysis increased to 2.15 ± 0.27%, remaining comparable to that observed with the liposomal control without **IL** (2.04 ± 0.27%) and the milli-Q control (1.87 ± 0.09%) (Figure 9). Statistical analysis showed no significant effect of the tested formulation on hemolysis (*p* = 0.6571) and no significant interaction between formulation and incubation time (*p* = 0.8088), whereas a significant overall effect of incubation time was observed (*p* = 0.0033; Figure S24, Supplementary Materials). These results indicate that removal of the **IL** fraction suspended in the aqueous phase did not result in an increase in hemolysis, suggesting that **IL** associated with the liposomal fraction did not substantially increase hemolysis of human erythrocytes under the conditions tested.

In summary, both CDFs and ULF exhibited low hemolytic activity toward human erythrocytes under the conditions tested. Even at the highest **IL** concentration tested (20 mol%), hemolysis remained below approximately 2.5% after 24 h of incubation. Notably, removal of the aqueous, non-liposomal **IL** fraction did not significantly affect the hemolytic response, as the ULF showed a hemolytic activity comparable to that of the corresponding CDF formulation and control liposomes. These findings indicate that the low hemolytic activity of the DPPC/cholesterol liposomal system was maintained after **IL** incorporation.

The hemolytic activity observed for both CDF and ULF containing **IL** was comparable to that previously observed for free **IL** in studies using human red blood cells. In our previous study [25], free **IL** also demonstrated low hemolytic activity over a wider concentration range of 0–200 µM and after a prolonged incubation period of 2–72 h. These findings indicate that the incorporation of **IL** did not substantially affect the low hemolytic activity of the DPPC/cholesterol liposomal system.

## 3. Materials and Methods

### 3.1. Chemicals

1,2-Dipalmitoyl-*sn*-glycero-3-phosphocholine (DPPC) was purchased from Avanti Polar Lipids (Delfzijl, The Netherlands). Cholesterol and merocyanine 540 (MC540) were purchased from Sigma-Aldrich^®^ (St. Louis, MO, USA). The fluorescent probes 1,6-diphenyl-1,3,5-hexatriene (DPH) and 1-[6-(dimethylamino)naphthalen-2-yl]dodecan-1-one (Laurdan) were purchased from Molecular Probes (Eugene, OR, USA). Chloroform, dichloromethane, acetone, disodium hydrogen phosphate dodecahydrate (Na_2_HPO_4_·12H_2_O), sodium dihydrogen phosphate monohydrate (NaH_2_PO_4_·H_2_O), and magnesium sulfate (MgSO_4_) were purchased from Chempur (Piekary Śląskie, Poland).

The phosphate buffer (PBS, pH 7.4) was prepared using Na_2_HPO_4_·12H_2_O and NaH_2_PO_4_·H_2_O.

### 3.2. Liposome Preparation

Liposomal formulations consisting of DPPC, cholesterol, and the test compound **IL** were prepared by thin-film hydration. A control formulation containing DPPC and cholesterol without **IL** was prepared in parallel. **IL** was included in the formulations at four different molar ratios, corresponding to 2, 5, 10, and 20 mol% relative to the total amount of DPPC and cholesterol. The molar ratio of DPPC to cholesterol was kept constant at 4:1 in all formulations, while the amount of **IL** was varied to obtain the desired molar ratio.

DPPC, cholesterol, and **IL** were dissolved in chloroform and mixed to obtain the desired formulations. The organic solvent was then removed by evaporation under a nitrogen stream, yielding a uniform thin lipid film, which was further dried under vacuum for 2 h to remove residual solvent.

The dried lipid films were hydrated with 1 mL of milli-Q water or phosphate buffer (pH 7.4). Hydration was performed at approximately 50 °C, above the main phase transition temperature of DPPC, to facilitate lipid hydration and bilayer formation. After hydration, the samples were vortexed until a homogeneous, milky suspension was obtained.

The resulting hydrated liposome suspensions were sonicated at approximately 50 °C for 1 min. Subsequently, all liposome suspensions were extruded using a Lipex^®^ extruder (Evonik, Vancouver, BC, Canada) equipped with 100 nm polycarbonate membranes (Avanti Research™, supplied by Sigma-Aldrich^®^, St. Louis, MO, USA) at RT. The suspensions were passed through the membrane for a total of eight extrusion cycles to obtain liposomal formulations with a more homogeneous particle size distribution.

For samples intended for subsequent physicochemical and spectroscopic analyses, the final volume of the liposomal suspensions was maintained at 1 mL. These analyses were performed only for the control formulation (DPPC/chol without **IL**) and the sample containing the highest **IL** concentration (20 mol%), both prepared by hydration with milli-Q water. Thus, samples prepared by hydration with either milli-Q water or PBS (pH 7.4) were subjected to DLS/ELS and hemolytic activity analyses.

### 3.3. Physicochemical Characterization of **IL**-Modified Liposomes by Dynamic Light Scattering (DLS)

The physicochemical properties and stability of the liposomal formulations were assessed using dynamic light scattering (DLS) and electrophoretic light scattering (ELS). The analysis included all **IL**-containing liposomal formulations (2, 5, 10, and 20 mol%), as well as the corresponding liposomes without **IL** used as controls. Liposomes hydrated with milli-Q water or buffer (PBS, pH 7.4) were characterized for mean hydrodynamic diameter, polydispersity index (PDI), and zeta potential.

For DLS/ELS measurements, the liposomal formulations were further diluted to a final total lipid concentration of 0.13 mg/mL, using milli-Q water or PBS (pH 7.4) as the appropriate medium. Following dilution, the final concentrations of **IL** in the 2, 5, 10, and 20 mol% formulations were 1.45, 3.62, 7.24, and 14.48 µg/mL, respectively.

Measurements were performed at room temperature using a Zetasizer Nano ZS (Malvern Instruments, Malvern, UK). Samples stored at 4 °C were allowed to equilibrate to RT for 2 h before measurements. The mean hydrodynamic diameter, PDI, and zeta potential were determined after liposome preparation (2 h) and after 48 h, one week, two weeks, and one month of storage. After preparation, liposome samples were stored at 4 °C or RT, protected from light. Both milli-Q- and PBS-hydrated samples were evaluated under both storage conditions. Because extrusion was performed at RT, 2 h measurements were performed only for samples stored at RT; samples intended for storage at 4 °C were first measured after 48 h of refrigerated storage. The same samples were used for measurements at all subsequent time points, without preparing new samples before each measurement.

### 3.4. Determination of Loading Efficiency (LE%) by Gas Chromatography (GC)

The loading efficiency of **IL** in liposomes was determined by ultracentrifugation, followed by extraction of the compound not incorporated in the liposomal fraction and quantitative analysis by gas chromatography. Control liposomes without **IL** and a liposome formulation with the highest **IL** concentration (20 mol%) were tested for this analysis. The liposomes were prepared according to the procedure described in Section 3.2, the samples for LE% determination were prepared only in milli-Q water.

After preparation, the liposome samples were ultracentrifuged in a Himac CS150NX ultracentrifuge (Eppendorf Group, Hitachinaka, Ibaraki, Japan) at 125,000× *g* for 2 h at 4 °C. After centrifugation, the aqueous supernatant was carefully collected, leaving the liposome pellet at the bottom of the centrifuge tube intact. The supernatant, containing the fraction of **IL** not incorporated into liposomes, was subjected to liquid–liquid extraction with 3 mL of dichloromethane. This extraction was repeated three times to maximize the recovery of free **IL** from the aqueous phase. The combined organic phases were dried over MgSO_4_, and the solvent was then evaporated to dryness using a rotary evaporator. The remaining **IL** was dissolved in 1 mL of acetone and quantified by GC.

GC analysis was performed using a 7890N gas chromatograph (Agilent Technologies, Santa Clara, CA, USA) equipped with an autosampler, a split injector, and a flame ionization detector (FID). Analysis was performed using an HP-1 fused silica capillary column (30 m × 0.25 mm inner diameter, 0.25 μm film thickness; Agilent Technologies, Santa Clara, CA, USA). Hydrogen was used as the carrier gas at a constant flow rate of 1.0 mL/min. Samples (1 μL) were injected in a 20:1 split ratio. The injector temperature was set to 300 °C. The oven temperature was programmed from 100 °C to 200 °C at a rate of 20 °C/min, followed by an increase to 300 °C at 10 °C/min, with a final hold at 300 °C for 1 min. The total analysis time was 16 min.

**IL** quantification was performed using a calibration curve prepared from **IL** standard solutions over the concentration range of 0–2 mg/mL. The calibration curve was constructed by plotting peak area versus the corresponding **IL** concentration and demonstrated good linearity over the tested concentration range (R^2^ = 0.9949). The resulting equation was used to calculate the concentration and amount of **IL** not incorporated into the liposomal fraction and recovered from the aqueous phase after ultracentrifugation.

Loading efficiency was calculated according to the following equation:

$$LE\%=\;\frac{M_{total\;}-\;M_{free}}{M_{total}}\times 100\%$$

where M*_total_* is the initial amount of **IL** used to prepare liposomes, and M*_free_* is the amount of free **IL** determined in the aqueous supernatant by GC.

The analysis was performed using three independently prepared liposome samples (*n* = 3), and the loading efficiency was expressed as the mean ± standard deviation. A liposomal DPPC/cholesterol formulation without **IL** was used as a control to confirm the absence of signals indicating the presence of **IL** in the tested formulations.

The recovery efficiency of the extraction procedure was additionally verified by subjecting a known amount of **IL** to the same extraction protocol described above. The recovery was calculated as the percentage of the recovered amount relative to the initial amount of **IL**.

### 3.5. Evaluation of Bilayer Properties in **IL**-Loaded Liposomes

#### 3.5.1. Fluorescence-Based Assessment of Liposomal Bilayer Properties

The effect of **IL** on lipid membrane fluidity and lipid packing in a DPPC/cholesterol liposome formulation was studied using the fluorimetric method previously described by Dunal et al. [26], with minor modifications. Three fluorescent probes were used to assess changes in membrane fluidity and lipid packing: MC540, Laurdan, and DPH, which localize in different regions of the lipid bilayer.

Two liposome formulations were analysed: DPPC/chol liposomes without **IL**, used as a control, and DPPC/chol liposomes containing **IL** at 20 mol% relative to the total amount of DPPC and cholesterol.

For the fluorescence assays, liposomes were prepared according to the procedure described in Section 3.2. The liposome formulations were incubated with the corresponding fluorescent probes at final concentrations of 0.13 mg/mL for liposomes and 1.3 µM for the fluorescent probes. For the formulation containing **IL** at 20 mol% relative to the total amount of DPPC and cholesterol, the final **IL** concentration was 14.48 µg/mL. The samples were incubated at 37 °C for 45 min in the dark.

Fluorescence measurements were performed over a temperature range of 25–51 °C using a Cary Eclipse fluorescence spectrophotometer (Varian, San Diego, CA, USA) or a FluoTime 300 fluorescence spectrometer (PicoQuant GmbH, Berlin, Germany). All fluorescence measurements were performed using three independently prepared samples for each liposome formulation (*n* = 3).

For MC540, fluorescence emission spectra were recorded in the wavelength range from 560 to 650 nm, with the maximum fluorescence intensity observed at approximately λ_em_ = 587 nm. The generalized polarization (GP) of Laurdan fluorescence and the fluorescence anisotropy (r) of DPH were calculated as previously described [26].

#### 3.5.2. Attenuated Total Reflectance Fourier Transform Infrared Spectroscopy (ATR-FTIR)

The ATR-FTIR method was based on the method described by Dunal et al. [26], with minor modifications.

Two liposome formulations were analysed: DPPC/chol liposome formulation without **IL**, used as a control, and liposomes formulated with **IL** at 20 mol% relative to the total amount of DPPC and cholesterol. The liposomes were prepared according to the procedure described in Section 3.2. The **IL**-containing liposome sample was concentrated tenfold, resulting in a final **IL** concentration of 0.1448 mg/mL. DPPC/chol liposomes and DPPC/chol liposomes containing 20 mol% of **IL** were applied directly onto the ZnSe ATR crystal (Thermo Fisher Scientific, Waltham, MA, USA). Subsequently, the samples were dehydrated for approximately 20 min.

Spectral measurements were performed at three temperatures: 25 °C, 37 °C, and 51 °C, using a Nicolet 6700 FT-IR spectrometer (Thermo Fisher Scientific, Waltham, MA, USA) equipped with a ZnSe ATR crystal. At each temperature, spectra were recorded in triplicate, and individual spectra were generated by averaging 128 scans at a resolution of 2 cm^−1^ over the spectral range of 700–4000 cm^−1^. Background spectra were recorded using a clean ZnSe ATR crystal prior to sample measurements.

All measurements were performed using a thermostatically controlled ATR accessory to maintain the required measurement temperature. Data were analysed using OMNIC 8.3.103 software (Thermo Nicolet, Thermo Fisher Scientific, Waltham, MA, USA). Measurements were performed in three independent experiments (*n* = 3), using two independent ATR plates for each experiment.

### 3.6. Hemolytic Activity of Liposomal Formulations

#### 3.6.1. Hemolytic Activity of Crude Dispersion Fractions (CDFs)

The hemolytic activity of CDFs was assessed using human red blood cells according to the method described by Dunal et al. [25], with minor modifications.

Red blood cells were obtained from healthy donors through the Blood Donation and Blood Treatment Center in Wrocław. In accordance with applicable Polish regulations, the use of red blood cells obtained from blood donation centers for experimental purposes does not require separate ethical approval.

The collected red blood cells were gently resuspended and washed three times with a cold isotonic saline buffer. After the final washing step, the red blood cell suspension was adjusted to a hematocrit of 12%.

Hemolytic activity was assessed for CDFs containing **IL** at concentrations of 2, 5, 10, and 20 mol% relative to the total amount of DPPC and cholesterol, as well as for the **IL**-free DPPC/cholesterol liposomes used as a control. All liposome preparations were prepared as described in Section 3.2 by hydration with milli-Q water.

For the hemolysis assay, each liposome preparation was combined with PBS (pH 7.4) to obtain a final lipid concentration of 0.0266 mg/mL, with a final sample volume of 1 mL and a red blood cell hematocrit of 1.2%. The final concentrations of **IL** in the 2, 5, 10, and 20 mol% formulations were 0.29, 0.72, 1.45, and 2.90 µg/mL, respectively.

The positive control, corresponding to 100% hemolysis (A_100%_), was prepared by suspending red blood cells in a sufficient excess of milli-Q water to induce complete hypotonic lysis. All experimental conditions were tested in triplicate (*n* = 3).

Samples were incubated at 37 °C for 1 and 24 h under static conditions. After incubation, the samples were centrifuged at 5000 rpm for 15 min at room temperature. The resulting supernatants were carefully collected and transferred to 3 mL cuvettes. The absorbance of released hemoglobin was measured at 540 nm using a spectrophotometer Specord 40 (AnalytikJena, Jena, Germany).

Erythrocyte hemolysis was calculated according to the equation described previously [25].

#### 3.6.2. Hemolytic Activity of Loaded Ultracentrifuged Liposomal Fraction (ULF)

The hemolytic activity of ULF containing 20 mol% **IL** was assessed according to the procedure described in Section 3.6.1, with the following modification. Liposomal preparations were purified by ultracentrifugation as described in Section 3.4. After centrifugation, the supernatant containing **IL** not loaded into the liposomal fraction was removed, and the resulting liposome pellet was resuspended in 1 mL of milli-Q water. The suspension was vortex-mixed until homogeneous.

For the hemolysis assay, 100 µL of the resulting 1 mL liposome suspension was used for each sample. Three replicates (*n* = 3) were used to assess the hemolysis of ULF. The results were expressed as the mean ± standard deviation of three independent measurements.

All other experimental conditions, including the RBC concentration, sample composition, incubation conditions, centrifugation, absorbance measurements, and calculation of hemolysis, were the same as those described in Section 3.6.1.

### 3.7. Statistical Analysis

Analyses were performed using GraphPad Prism 8 software (GraphPad Software, San Diego, CA, USA). Prior to hypothesis testing, data distribution normality was assessed. Depending on the experimental design, data were analysed using two-way analysis of variance (ANOVA), followed by Dunnett’s multiple comparisons test for comparisons with the control group or Šídák’s multiple comparisons test for selected pairwise comparisons.

For analyses performed using Šídák’s or Dunnett’s test, statistical significance was denoted as follows: ns—not significant, * *p* < 0.05, ** *p* < 0.01, *** *p* < 0.001, and **** *p* < 0.0001.

## 4. Conclusions

The DPPC/chol liposomal formulation containing 20 mol% **IL** exhibited favorable physicochemical properties, a high loading efficiency, and low hemolytic activity under the conditions investigated. The selection of the DPPC/cholesterol formulation containing 20 mol% **IL** was based on its favorable physicochemical properties and high loading efficiency for **IL** while maintaining bilayer organization. Incorporation of **IL** into the lipid bilayer enabled its effective dispersion in the aqueous phase, while the presence of cholesterol contributed to the preservation of bilayer organization and stability.

Under the selected storage conditions (milli-Q water, 4 °C), **IL**-loaded liposomes maintained stable physicochemical properties, as demonstrated by relatively stable particle size, low PDI, and preserved negative zeta potential during one month of storage. The high loading efficiency (87.6 ± 7.8%) indicated that the majority of the initial **IL** was not recovered in the aqueous supernatant after ultracentrifugation, suggesting that it was associated with the liposomal fraction. However, the potential sedimentation of free **IL** during ultracentrifugation cannot be ruled out, which represents a technical limitation of this assay; thus, the reported LE% should be interpreted as an indirect estimate of **IL** loading. Future control experiments involving the ultracentrifugation of free **IL** in the absence of liposomes will help to quantify this contribution and refine the accuracy of the LE% calculation.

Fluorescence and ATR-FTIR analyses demonstrated that **IL** primarily interacts with the polar and interfacial regions of the bilayer while causing no substantial disruption of the hydrophobic acyl chain region. This localization is consistent with the amphiphilic and lipophilic character of **IL** and suggests that its association with the membrane occurs predominantly within the polar and interfacial region, while preserving the organization of the hydrophobic acyl chains. Finally, both CDFs and ULF **IL**-loaded liposomes exhibited low hemolytic activity, indicating that **IL** incorporation did not substantially increase hemolysis under the conditions tested.

Taken together, these findings provide a physicochemical characterization of **IL**-loaded DPPC/chol liposomes and demonstrate their favorable colloidal properties, **IL** loading efficiency, and membrane organization under the conditions investigated.

To establish the performance of these liposomes as a drug delivery system, further studies should be conducted. These should focus on evaluating the cytotoxicity of the developed liposomal formulation, characterizing the release profile of **IL** from DPPC/chol liposomes under physiological temperature and factors mimicking the tumor microenvironment, and assessing their stability under physiologically relevant conditions. Further optimization of the surface properties of the carrier, including through the incorporation of PEGylated lipids or other suitable surfactants, should also be considered in future studies.

## Figures and Tables

**Table 1:** Selected FTIR-ATR band frequencies of DPPC/chol liposomes in the presence of compound **IL** (20 mol%) at different temperatures. Data are presented as mean ± SD (*n* = 3).

Frequency [cm^−1^]	25 °C		37 °C		51 °C	
	DPPC/Chol	DPPC/Chol + IL	DPPC/Chol	DPPC/Chol + IL	DPPC/Chol	DPPC/Chol + IL
γ_r_(CH_2_)	721.32 ± 0.02	721.46 ± 0.02	721.21 ± 0.04	721.27 ± 0.01	721.01 ± 0.01	721.18 ± 0.01
ν_as_(P-(OC)_2_)	822.86 ± 0.03	823.71 ± 0.09	821.60 ± 0.19	822.01 ± 0.23	819.86 ± 0.11	820.88 ± 0.04
ν_s_(N-C)	926.39 ± 0.10	926.08 ± 0.06	925.91 ± 0.14	925.35 ± 0.02	925.44 ± 0.00	924.91 ± 0.05
ν_as_(N-C)	970.49 ± 0.11	970.29 ± 0.05	969.72 ± 0.13	969.51 ± 0.09	969.13 ± 0.02	969.11 ± 0.00
ν(C-O-P)	1066.00 ± 0.01	1065.33 ± 0.01	1066.39 ± 0.00	1065.58 ± 0.04	1066.76 ± 0.14	1065.65 ± 0.02
ν_s_(PO_2_^−^)	1088.59 ± 0.07	1088.65 ± 0.07	1089.71 ± 0.19	1089.42 ± 0.06	1090.38 ± 0.03	1089.77 ± 0.01
ν(C-O-C)	1171.02 ± 0.07	1172.08 ± 0.13	1169.95 ± 0.21	1171.12 ± 0.10	1169.32 ± 0.08	1170.77 ± 0.13
ν_as_(PO_2_^−^)	1231.97 ± 0.64	1231.21 ± 0.62	1241.65 ± 0.82	1240.47 ± 0.74	1243.15 ± 0.07	1241.98 ± 0.00
δ_s_(CH_3_)	1377.39 ± 0.02	1377.47 ± 0.03	1377.09 ± 0.05	1377.26 ± 0.03	1377.30 ± 0.18	1377.36 ± 0.17
δ(CH_2_)	1467.84 ± 0.02	1467.88 ± 0.01	1467.86 ± 0.03	1467.84 ± 0.03	1467.52 ± 0.11	1467.67 ± 0.06
ν(C=O)	1742.09 ± 0.02	1741.83 ± 0.06	1742.60 ± 0.12	1741.97 ± 0.15	1741.42 ± 0.21	1741.66 ± 0.27
	1733.59 ± 0.05	1733.72 ± 0.02	1733.64 ± 0.03	1733.71 ± 0.09	1733.37 ± 0.12	1733.59 ± 0.09
ν_s_(CH_2_)	2849.99 ± 0.03	2850.03 ± 0.03	2850.04 ± 0.02	2850.17 ± 0.01	2850.21 ± 0.01	2850.29 ± 0.05
ν_s_(CH_3_)	2871.10 ± 0.15	2870.50 ± 0.00	2870.30 ± 0.02	-	-	-
ν_as_(CH_2_)	2918.13 ± 0.09	2918.28 ± 0.01	2918.26 ± 0.38	2918.57 ± 0.06	2918.62 ± 0.01	2918.83 ± 0.13
ν_as_(CH_3_)	2956.30 ± 0.00	2956.21 ± 0.10	2956.34 ± 0.08	2956.28 ± 0.08	2956.45 ± 0.02	2956.35 ± 0.12

Vibrations: δ—bending, ν—stretching, γ—deformation; index: s—symmetric, as—asymmetric; and r—rocking.

## Data Availability

The datasets used and/or analysed during the current study are available from the corresponding author upon reasonable request.

## Supplementary Materials

The following supporting information can be downloaded at https://www.mdpi.com/article/10.3390/molecules31203756/s1, Figure S1: Chemical structures of 1,2-dipalmitoyl-*sn*-glycero-3-phosphocholine (DPPC) and cholesterol. Figure S2: Polydispersity index (PDI) of DPPC/chol liposomes containing 0, 2, 5, 10, and 20 mol% IL, measured after preparation (2 h) and after 48 h, one week, two weeks, and one month of storage. “0” means DPPC/chol liposomes without IL. Data are presented as mean ± standard deviation (*n* = 3). Figure S3: DLS particle size distribution profiles of DPPC/chol liposomes containing 0 and 20 mol% IL, hydrated with PBS (pH 7.4) and measured after preparation (2 h). (A) 0 mol% IL; (B) 20 mol% IL. “0” means DPPC/chol liposomes without IL. Three replicate measurements are shown for each formulation (red—replicate 1; green—replicate 2; blue—replicate 3). Figure S4: DLS particle size distribution profiles of DPPC/chol liposomes containing 0 and 20 mol% IL, hydrated with milli-Q water and measured after 48 h of storage. (A) DPPC/chol liposomes containing 0 mol% IL, stored at 4 °C; (B) DPPC/chol liposomes containing 20 mol% IL, stored at 4 °C; (C) DPPC/chol liposomes containing 0 mol% IL, stored at RT; (D) DPPC/chol liposomes containing 20 mol% IL, stored at RT. “0” means DPPC/chol liposomes without IL. Three replicate measurements are shown for each formulation (red—replicate 1; green—replicate 2; blue—replicate 3). Figure S5: DLS particle size distribution profiles of DPPC/chol liposomes containing 0 and 20 mol% IL, hydrated with PBS (pH 7.4) and measured after 48 h of storage. (A) DPPC/chol liposomes containing 0 mol% IL, stored at 4 °C; (B) DPPC/chol liposomes containing 20 mol% IL, stored at 4 °C; (C) DPPC/chol liposomes containing 0 mol% IL, stored at RT; (D) DPPC/chol liposomes containing 20 mol% IL, stored at RT. “0” means DPPC/chol liposomes without IL. Three replicate measurements are shown for each formulation (red—replicate 1; green—replicate 2; blue—replicate 3). Figure S6: DLS particle size distribution profiles of DPPC/chol liposomes containing 0 and 20 mol% IL, hydrated with milli-Q water and measured after 7 days of storage. (A) DPPC/chol liposomes containing 0 mol% IL, stored at 4 °C; (B) DPPC/chol liposomes containing 20 mol% IL, stored at 4 °C; (C) DPPC/chol liposomes containing 0 mol% IL, stored at RT; (D) DPPC/chol liposomes containing 20 mol% IL, stored at RT. “0” means DPPC/chol liposomes without IL. Three replicate measurements are shown for each formulation (red—replicate 1; green—replicate 2; blue—replicate 3). Figure S7: DLS particle size distribution profiles of DPPC/chol liposomes containing 0 and 20 mol% IL, hydrated with PBS (pH 7.4) and measured after 7 days of storage. (A) DPPC/chol liposomes containing 0 mol% IL, stored at 4 °C; (B) DPPC/chol liposomes containing 20 mol% IL, stored at 4 °C; (C) DPPC/chol liposomes containing 0 mol% IL, stored at RT; (D) DPPC/chol liposomes containing 20 mol% IL, stored at RT. “0” means DPPC/chol liposomes without IL. Three replicate measurements are shown for each formulation (red—replicate 1; green—replicate 2; blue—replicate 3). Figure S8: DLS particle size distribution profiles of DPPC/chol liposomes containing 0 and 20 mol% IL, hydrated with milli-Q water and measured after 14 days of storage. (A) DPPC/chol liposomes containing 0 mol% IL, stored at 4 °C; (B) DPPC/chol liposomes containing 20 mol% IL, stored at 4 °C; (C) DPPC/chol liposomes containing 0 mol% IL, stored at RT; (D) DPPC/chol liposomes containing 20 mol% IL, stored at RT. “0” means DPPC/chol liposomes without IL. Three replicate measurements are shown for each formulation (red—replicate 1; green—replicate 2; blue—replicate 3). Figure S9: DLS particle size distribution profiles of DPPC/chol liposomes containing 0 and 20 mol% IL, hydrated with PBS (pH 7.4) and measured after 14 days of storage. (A) DPPC/chol liposomes containing 0 mol% IL, stored at 4 °C; (B) DPPC/chol liposomes containing 20 mol% IL, stored at 4 °C; (C) DPPC/chol liposomes containing 0 mol% IL, stored at RT; (D) DPPC/chol liposomes containing 20 mol% IL, stored at RT. “0” means DPPC/chol liposomes without IL. Three replicate measurements are shown for each formulation (red—replicate 1; green—replicate 2; blue—replicate 3). Figure S10: DLS particle size distribution profiles of DPPC/chol liposomes containing 0 and 20 mol% IL, hydrated with milli-Q water and measured after one month of storage. (A) DPPC/chol liposomes containing 0 mol% IL, stored at 4 °C; (B) DPPC/chol liposomes containing 20 mol% IL, stored at 4 °C; (C) DPPC/chol liposomes containing 0 mol% IL, stored at RT; (D) DPPC/chol liposomes containing 20 mol% IL, stored at RT. “0” means DPPC/chol liposomes without IL. Three replicate measurements are shown for each formulation (red—replicate 1; green—replicate 2; blue—replicate 3). Figure S11: DLS particle size distribution profiles of DPPC/chol liposomes containing 0 and 20 mol% IL, hydrated with PBS (pH 7.4) and measured after one month of storage. (A) DPPC/chol liposomes containing 0 mol% IL, stored at 4 °C; (B) DPPC/chol liposomes containing 20 mol% IL, stored at 4 °C; (C) DPPC/chol liposomes containing 0 mol% IL, stored at RT; (D) DPPC/chol liposomes containing 20 mol% IL, stored at RT. “0” means DPPC/chol liposomes without IL. Three replicate measurements are shown for each formulation (red—replicate 1; green—replicate 2; blue—replicate 3). Figure S12: Hydrodynamic diameter of DPPC/chol liposomes containing 0, 2, 5, 10, and 20 mol% IL, measured after preparation (2 h) and after 48 h, one week, two weeks, and one month of storage. “0” means DPPC/chol liposomes without IL. Data are presented as mean ± standard deviation (*n* = 3). Figure S13: Zeta potential of DPPC/chol liposomes containing 0, 2, 5, 10, and 20 mol% IL, measured after preparation (2 h) and after 48 h, one week, two weeks, and one month of storage. “0” means DPPC/chol liposomes without IL. Data are presented as mean ± standard deviation (*n* = 3). Figure S14: Calibration curve for the quantitative determination of IL by GC. The curve was obtained using IL standard solutions in the concentration range of 0–2 mg/mL. Figure S15: Chemical structures of merocyanine 540 (MC540), 1-[6-(dimethylamino)naphthalen-2-yl]dodecan-1-one (Laurdan), and 1,6-diphenyl-1,3,5-hexatriene (DPH) used as fluorescent membrane probes. Figure S16: Statistical analysis of the data presented in Figure 5. Statistical analysis was performed using two-way analysis of variance (ANOVA), followed by Šídák’s multiple comparisons test. Data are presented as mean ± standard deviation (*n* = 3). Figure S17: Statistical analysis of the data presented in Figure 6. Statistical analysis was performed using two-way analysis of variance (ANOVA), followed by Šídák’s multiple comparisons test. Data are presented as mean ± standard deviation (*n* = 3). Figure S18: Statistical analysis of the data presented in Figure 7. Statistical analysis was performed using two-way analysis of variance (ANOVA), followed by Šídák’s multiple comparisons test. Data are presented as mean ± standard deviation (*n* = 3). Figure S19: Raw fluorescence excitation spectra of the DPH recorded at two temperatures for DPPC/chol liposomes and DPPC/chol liposomes with the addition of 20 mol% IL. Figure S20: Raw polarized fluorescence intensity component spectra (I*_VV_*, I*_VH_*, I*_HV_*, I*_HH_*) of the DPH probe recorded at three temperatures for pure liposomes DPPC/chol and liposomes with the addition of 20 mol% IL. I*_VV_*—vertically polarized excitation/vertically polarized emission; I*_VH_*—vertically polarized excitation/horizontally polarized emission; I*_HV_*—horizontally polarized excitation/vertically polarized emission; I*_HH_*—horizontally polarized excitation/horizontally polarized emission. Figure S21: ATR-FTIR spectra of dehydrated DPPC/chol liposomes and liposomes in the presence of IL (20 mol%) at different temperatures. The main diagnostic regions are indicated: (A) C-H stretching vibrations of the hydrocarbon chains (2800–3000 cm^−1^), (B) carbonyl group stretching vibrations (1700–1750 cm^−1^), and (C) phosphate group vibrations (1080–1260 cm^−1^). Figure S22: Deconvolution of the carbonyl group (C=O) stretching vibration band in the ATR-FTIR spectrum measured at 25 °C for (A) DPPC/chol liposomes and (B) liposomes with the addition of 20 mol% IL. Line designations: green/cyan—experimental spectrum, red—fitted cumulative curve, purple—component of free C=O groups (~1742 cm^−1^); blue—component of hydrogen-bonded “C=O” groups (~1730 cm^−1^). Figure S23: Statistical analysis of the data presented in Figure 8. Statistical analysis was performed using two-way analysis of variance (ANOVA), followed by Dunnett’s multiple comparisons test for comparisons with the control group. Data are presented as mean ± standard deviation (*n* = 3). Figure S24: Statistical analysis of the data presented in Figure 9. Statistical analysis was performed using two-way analysis of variance (ANOVA), followed by Dunnett’s multiple comparisons test for comparisons with the control group. Data are presented as mean ± standard deviation (*n* = 3).
