# Supplementary material for: Physicochemical Characterization of Liposomal Bilayer Models Loaded with Trans-4-(4′-hydroxy-3′-methoxyphenyl)-5-(1-iodoethyl)dihydrofuran-2-one
Source: Molecules. 2026 Oct 19;31(20):3756. doi: 10.3390/molecules31203756 (PMC13707891; doi:10.3390/molecules31203756)
Supplement: Supplementary file 1 [file molecules-31-03756-s001.zip › molecules-4588855-supplementary.pdf]

Supplementary Information

Physicochemical Characterization of Liposomal Bilayer Models Loaded with *Trans*-4-(4′-hydroxy-3′-methoxyphenyl)-5-(1-iodoethyl)dihydrofuran-2-one

Anna Dunal[1,*], Aleksandra Włoch[2], Witold Gładkowski[1], Magdalena Domańska[3] and Hanna Pruchnik[2,*]

[1] Department of Food Chemistry and Biocatalysis, Faculty of Biotechnology and Food Sciences , Wrocław University of Environmental and Life Sciences, Norwida 25, 50-375 Wrocław, Poland; witold.gladkowski@upwr.edu.pl (W.G);

[2] Department of Physics and Biophysics, Faculty of Biotechnology and Food Sciences, Wrocław University of Environmental and Life Sciences, Norwida 25, 50-375 Wrocław, Poland; aleksandra.wloch@upwr.edu.pl (A.W.);

[3] Institute of Environmental Engineering, Faculty of Environmental Engineering and Geodesy, Wrocław University of Environmental and Life Sciences, pl. Grunwaldzki 24, Wrocław 50-363, Poland magdalena.domanska@upwr.edu.pl (M.D.);

* Correspondence: anna.dunal@upwr.edu.pl (A.D.), hanna.pruchnik@upwr.edu.pl (H.P.);

Table of content

	Page
**Figure S1.** Chemical structures of 1,2-dipalmitoyl-*sn*-glycero-3-phosphocholine (DPPC) and cholesterol.	4
**Figure S2.** Polydispersity index (PDI) of DPPC/chol liposomes containing 0, 2, 5, 10, and 20 mol% **IL**, measured after preparation (2 h) and after 48 h, one week, two weeks, and one month of storage. "0" means DPPC/chol liposomes without **IL**. Data are presented as mean ± standard deviation (n = 3).	4
**Figure S3.** DLS particle size distribution profiles of DPPC/chol liposomes containing 0 and 20 mol% **IL**, hydrated with PBS (pH 7.4) and measured after preparation (2 h). (**A**) 0 mol% **IL**; (**B**) 20 mol% **IL**. "0" means DPPC/chol liposomes without **IL**. Three replicate measurements are shown for each formulation (red - replicate 1; green - replicate 2; blue - replicate 3).	5
**Figure S4.** DLS particle size distribution profiles of DPPC/chol liposomes containing 0 and 20 mol% **IL**, hydrated with milli-Q water and measured after 48 h of storage. (**A**) DPPC/chol liposomes containing 0 mol% **IL**, stored at 4 °C; (**B**) DPPC/chol liposomes containing 20 mol% **IL**, stored at 4 °C; (**C**) DPPC/chol liposomes containing 0 mol% **IL**, stored at RT; (**D**) DPPC/chol liposomes containing 20 mol% **IL**, stored at RT. "0" means DPPC/chol liposomes without **IL**. Three replicate measurements are shown for each formulation (red - replicate 1; green - replicate 2; blue - replicate 3).	5
**Figure S5.** DLS particle size distribution profiles of DPPC/chol liposomes containing 0 and 20 mol% **IL**, hydrated with PBS (pH 7.4) and measured after 48 h of storage. (**A**) DPPC/chol liposomes containing 0 mol% **IL**, stored at 4 °C; (**B**) DPPC/chol liposomes containing 20 mol% **IL**, stored at 4 °C; (**C**) DPPC/chol liposomes containing 0 mol% **IL**, stored at RT; (**D**) DPPC/chol liposomes containing 20 mol% **IL**, stored at RT. "0" means DPPC/chol liposomes without **IL**. Three replicate measurements are shown for each formulation (red - replicate 1; green - replicate 2; blue - replicate 3).	6
**Figure S6.** DLS particle size distribution profiles of DPPC/chol liposomes containing 0 and 20 mol% **IL**, hydrated with milli-Q water and measured after 7 days of storage. (**A**) DPPC/chol liposomes containing 0 mol% **IL**, stored at 4 °C; (**B**) DPPC/chol liposomes containing 20 mol% **IL**, stored at 4 °C; (**C**) DPPC/chol liposomes containing 0 mol% **IL**, stored at RT; (**D**) DPPC/chol liposomes containing 20 mol% **IL**, stored at RT. "0" means DPPC/chol liposomes without **IL**. Three replicate measurements are shown for each formulation (red - replicate 1; green - replicate 2; blue - replicate 3).	6
**Figure S7.** DLS particle size distribution profiles of DPPC/chol liposomes containing 0 and 20 mol% **IL**, hydrated with PBS (pH 7.4) and measured after 7 days of storage. (**A**) DPPC/chol liposomes containing 0 mol% **IL**, stored at 4 °C; (**B**) DPPC/chol liposomes containing 20 mol% **IL**, stored at 4 °C; (**C**) DPPC/chol liposomes containing 0 mol% **IL**, stored at RT; (**D**) DPPC/chol liposomes containing 20 mol% **IL**, stored at RT. "0" means DPPC/chol liposomes without **IL**. Three replicate measurements are shown for each formulation (red - replicate 1; green - replicate 2; blue - replicate 3).	7
**Figure S8.** DLS particle size distribution profiles of DPPC/chol liposomes containing 0 and 20 mol% **IL**, hydrated with milli-Q water and measured after 14 days of storage. (**A**) DPPC/chol liposomes containing 0 mol% **IL**, stored at 4 °C; (**B**) DPPC/chol liposomes containing 20 mol% **IL**, stored at 4 °C; (**C**) DPPC/chol liposomes containing 0 mol% **IL**, stored at RT; (**D**) DPPC/chol liposomes containing 20 mol% **IL**, stored at RT. "0" means DPPC/chol liposomes without **IL**. Three replicate measurements are shown for each formulation (red - replicate 1; green - replicate 2; blue - replicate 3).	7
**Figure S9.** DLS particle size distribution profiles of DPPC/chol liposomes containing 0 and 20 mol% **IL**, hydrated with PBS (pH 7.4) and measured after 14 days of storage. (**A**) DPPC/chol liposomes containing 0 mol% **IL**, stored at 4 °C; (**B**) DPPC/chol liposomes containing 20 mol% **IL**, stored at 4 °C; (**C**) DPPC/chol liposomes containing 0 mol% **IL**, stored at RT; (**D**) DPPC/chol liposomes containing 20 mol% **IL**, stored at RT. "0" means DPPC/chol liposomes without **IL**. Three replicate measurements are shown for each formulation (red - replicate 1; green - replicate 2; blue - replicate 3).	8
**Figure S10.** DLS particle size distribution profiles of DPPC/chol liposomes containing 0 and 20 mol% **IL**, hydrated with milli-Q water and measured after one month of storage. (**A**) DPPC/chol liposomes containing 0 mol% **IL**, stored at 4 °C; (**B**) DPPC/chol liposomes containing 20 mol% **IL**, stored at 4 °C; (**C**) DPPC/chol liposomes containing 0 mol% **IL**, stored at RT; (**D**) DPPC/chol liposomes containing 20 mol% **IL**, stored at RT. "0" means DPPC/chol liposomes without **IL**. Three replicate measurements are shown for each formulation (red - replicate 1; green - replicate 2; blue - replicate 3).	8
**Figure S11.** DLS particle size distribution profiles of DPPC/chol liposomes containing 0 and 20 mol% **IL**, hydrated with PBS (pH 7.4) and measured after one month of storage. (**A**) DPPC/chol liposomes containing 0 mol% **IL**, stored at 4 °C; (**B**) DPPC/chol liposomes containing 20 mol% **IL**, stored at 4 °C; (**C**) DPPC/chol liposomes containing 0 mol% **IL**, stored at RT; (**D**) DPPC/chol liposomes containing 20 mol% **IL**, stored at RT. "0" means DPPC/chol liposomes without **IL**. Three replicate measurements are shown for each formulation (red - replicate 1; green - replicate 2; blue - replicate 3).	9
**Figure S12.** Hydrodynamic diameter of DPPC/chol liposomes containing 0, 2, 5, 10, and 20 mol% **IL**, measured after preparation (2 h) and after 48 h, one week, two weeks, and one month of storage. "0" means DPPC/chol liposomes without **IL**. Data are presented as mean ± standard deviation (n = 3).	9

**Figure S13**. Zeta potential of DPPC/chol liposomes containing 0, 2, 5, 10, and 20 mol% **IL**, measured after preparation (2 h) and after 48 h, one week, two weeks, and one month of storage. "0" means DPPC/chol liposomes without **IL**. Data are presented as mean ± standard deviation (*n* = 3).	10
**Figure S14.** Calibration curve for the quantitative determination of **IL** by GC. The curve was obtained using **IL** standard solutions in the concentration range of 0-2 mg/mL.	10
**Figure S15.** Chemical structures of merocyanine 540 (MC540), 1-[6-(dimethylamino)naphthalen-2-yl]dodecan-1-one (Laurdan), and 1,6-diphenyl-1,3,5-hexatriene (DPH) used as fluorescent membrane probes.	11
**Figure S16**. Statistical analysis of the data presented in **Figure 5**. Statistical analysis was performed using two-way analysis of variance (ANOVA), followed by Šídák's multiple comparisons test. Data are presented as mean ± standard deviation (*n* = 3).	11
**Figure S17**. Statistical analysis of the data presented in **Figure 6**. Statistical analysis was performed using two-way analysis of variance (ANOVA), followed by Šídák's multiple comparisons test. Data are presented as mean ± standard deviation (*n* = 3).	12
**Figure S18**. Statistical analysis of the data presented in **Figure 7**. Statistical analysis was performed using two-way analysis of variance (ANOVA), followed by Šídák's multiple comparisons test. Data are presented as mean ± standard deviation (*n* = 3).	12
**Figure S19.** Raw fluorescence excitation spectra of the DPH recorded at two temperatures for DPPC/chol liposomes and DPPC/chol liposomes with the addition of 20 mol% **IL**.	13
**Figure S20.** Raw polarized fluorescence intensity components spectra (I_{VV}, I_{VH}, I_{HV}, I_{HH}) of the DPH probe recorded at three temperatures for: pure liposomes DPPC/chol and liposomes with the addition of 20 mol% **IL**. I_{VV} - vertically polarized excitation/vertically polarized emission; I_{VH} - vertically polarized excitation/horizontally polarized emission; I_{HV} - horizontally polarized excitation/vertically polarized emission; I_{HH} - horizontally polarized excitation/horizontally polarized emission.	14
**Figure S21**. ATR-FTIR spectra of dehydrated DPPC/chol liposomes and liposomes in the presence of **IL** (20 mol%) at different temperatures. The main diagnostic regions are indicated: (**A**) C-H stretching vibrations of the hydrocarbon chains (2800-3000 cm^{-1}), (**B**) carbonyl group stretching vibrations (1700-1750 cm^{-1}), and (**C**) phosphate group vibrations (1080-1260 cm^{-1}).	15
**Figure S22.** Deconvolution of the carbonyl group (C=O) stretching vibration band in the ATR-FTIR spectrum measured at 25 °C for: (**A**) DPPC/chol liposomes and (**B**) liposomes with the addition of 20 mol% **IL**. Line designations: green/cyan - experimental spectrum, red - fitted cumulative curve, purple - component of free C=O groups (~1742 cm^{-1}); blue - component of hydrogen-bonded C=O groups (~1730 cm^{-1}).	15
**Figure S23.** Statistical analysis of the data presented in **Figure 8.** Statistical analysis was performed using two-way analysis of variance (ANOVA), followed by Dunnett's multiple comparisons test for comparisons with the control group. Data are presented as mean ± standard deviation (*n* = 3).	16
**Figure S24.** Statistical analysis of the data presented in **Figure 9**. Statistical analysis was performed using two-way analysis of variance (ANOVA), followed by Dunnett's multiple comparisons test for comparisons with the control group. Data are presented as mean ± standard deviation (*n* = 3).	16

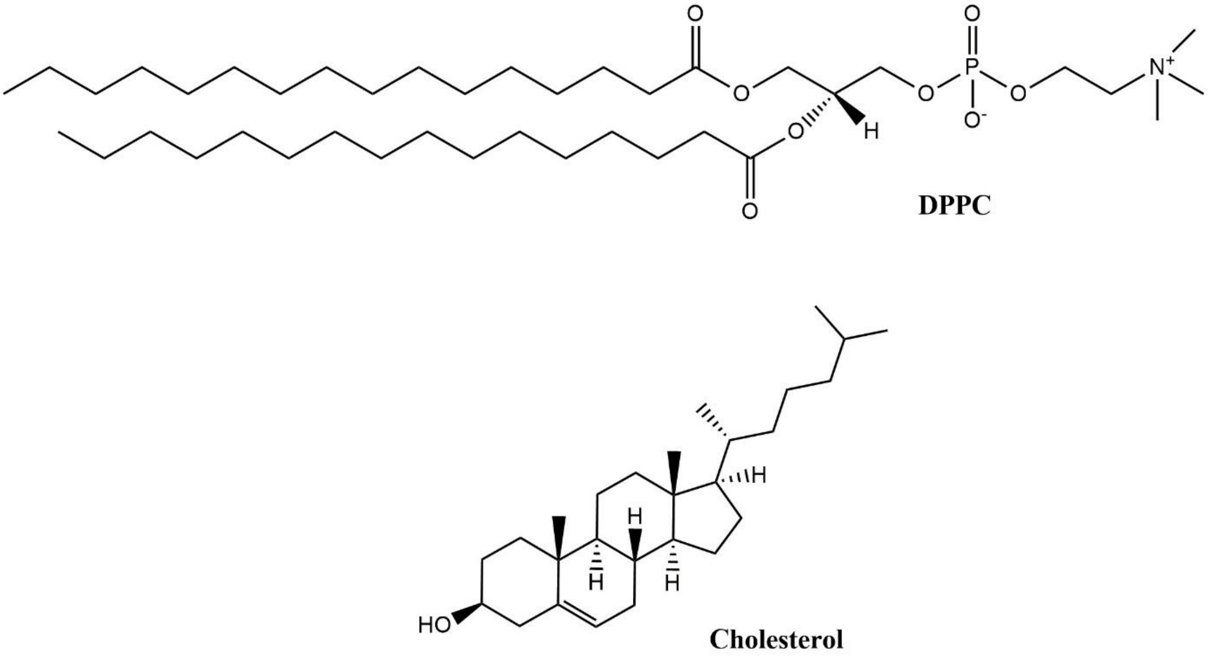

Figure S1. Chemical structures of 1,2-dipalmitoyl-*sn*-glycero-3-phosphocholine (DPPC) and cholesterol.

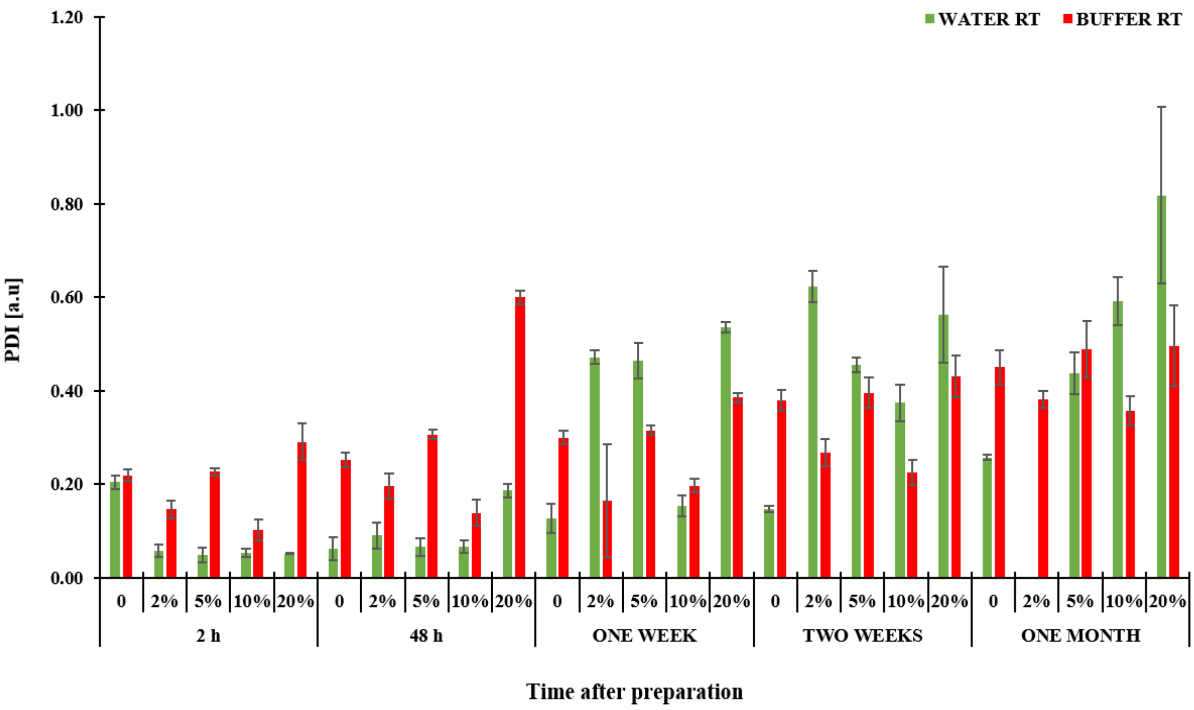

Figure S2. Polydispersity index (PDI) of DPPC/chol liposomes containing 0, 2, 5, 10, and 20 mol% **IL**, measured after preparation (2 h) and after 48 h, one week, two weeks, and one month of storage. "0" means DPPC/chol liposomes without **IL**. Data are presented as mean ± standard deviation (n = 3).

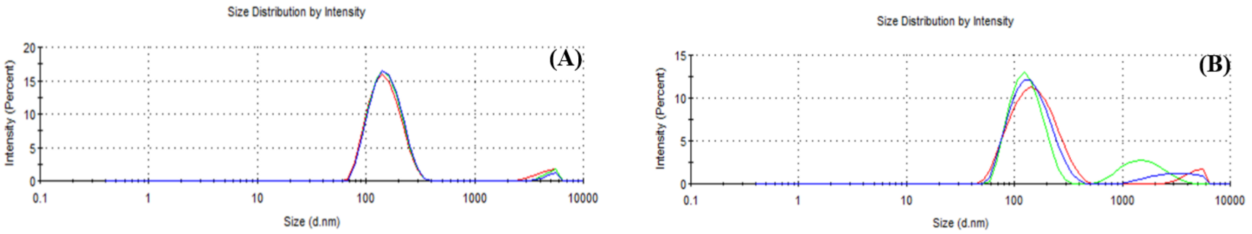

Figure S3. DLS particle size distribution profiles of DPPC/chol liposomes containing 0 and 20 mol% **IL**, hydrated with PBS (pH 7.4) and measured after preparation (2 h). (**A**) 0 mol% **IL**; (**B**) 20 mol% **IL**. "0" means DPPC/chol liposomes without **IL**. Three replicate measurements are shown for each formulation (red - replicate 1; green - replicate 2; blue - replicate 3).

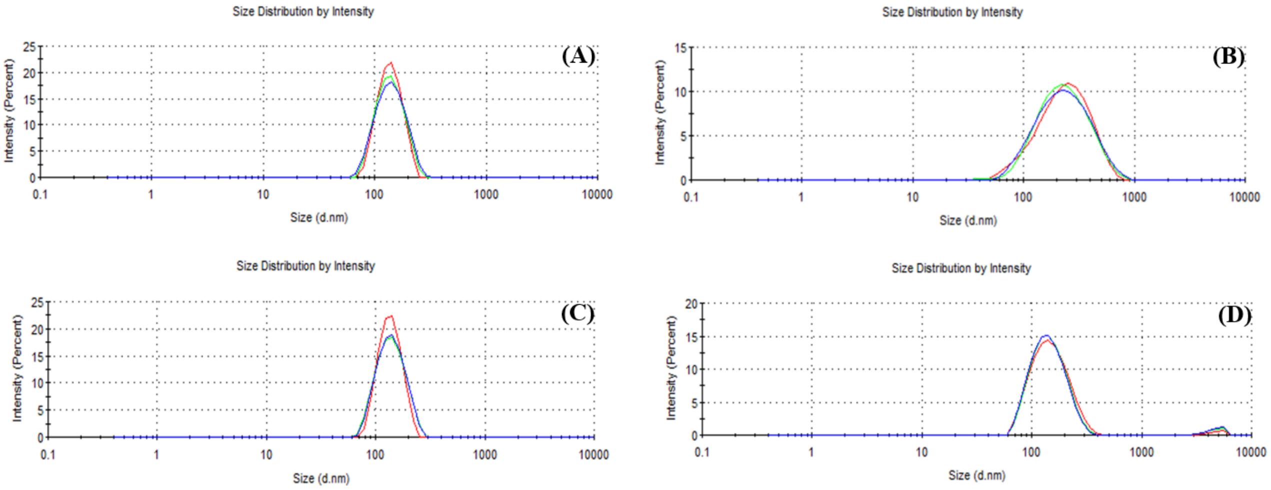

Figure S4. DLS particle size distribution profiles of DPPC/chol liposomes containing 0 and 20 mol% **IL**, hydrated with milli-Q water and measured after 48 h of storage. (**A**) DPPC/chol liposomes containing 0 mol% **IL**, stored at 4 °C; (**B**) DPPC/chol liposomes containing 20 mol% **IL**, stored at 4 °C; (**C**) DPPC/chol liposomes containing 0 mol% **IL**, stored at RT; (**D**) DPPC/chol liposomes containing 20 mol% **IL**, stored at RT. "0" means DPPC/chol liposomes without **IL**. Three replicate measurements are shown for each formulation (red - replicate 1; green - replicate 2; blue - replicate 3).

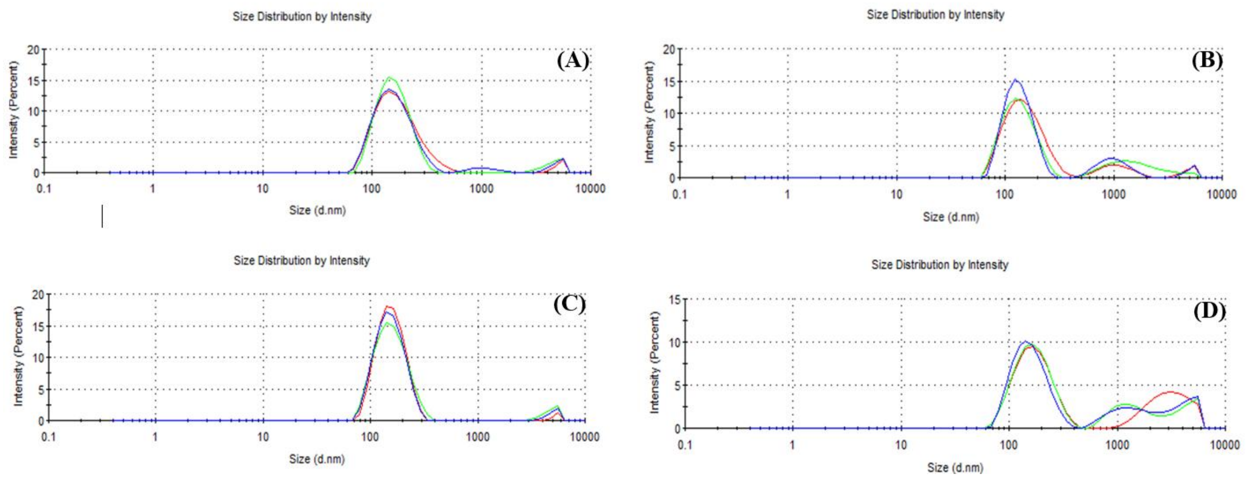

Figure S5. DLS particle size distribution profiles of DPPC/chol liposomes containing 0 and 20 mol% **IL**, hydrated with PBS (pH 7.4) and measured after 48 h of storage. (**A**) DPPC/chol liposomes containing 0 mol% **IL**, stored at 4 °C; (**B**) DPPC/chol liposomes containing 20 mol% **IL**, stored at 4 °C; (**C**) DPPC/chol liposomes containing 0 mol% **IL**, stored at RT; (**D**) DPPC/chol liposomes containing 20 mol% **IL**, stored at RT. "0" means DPPC/chol liposomes without **IL**. Three replicate measurements are shown for each formulation (red - replicate 1; green - replicate 2; blue - replicate 3).

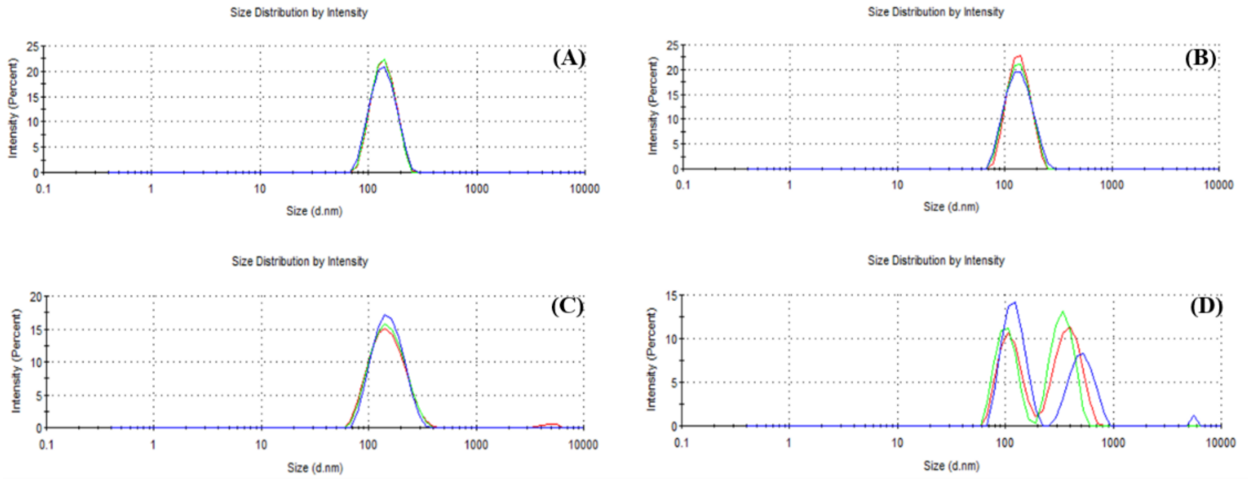

Figure S6. DLS particle size distribution profiles of DPPC/chol liposomes containing 0 and 20 mol% **IL**, hydrated with milli-Q water and measured after 7 days of storage. (**A**) DPPC/chol liposomes containing 0 mol% **IL**, stored at 4 °C; (**B**) DPPC/chol liposomes containing 20 mol% **IL**, stored at 4 °C; (**C**) DPPC/chol liposomes containing 0 mol% **IL**, stored at RT; (**D**) DPPC/chol liposomes containing 20 mol% **IL**, stored at RT. "0" means DPPC/chol liposomes without **IL**. Three replicate measurements are shown for each formulation (red - replicate 1; green - replicate 2; blue - replicate 3).

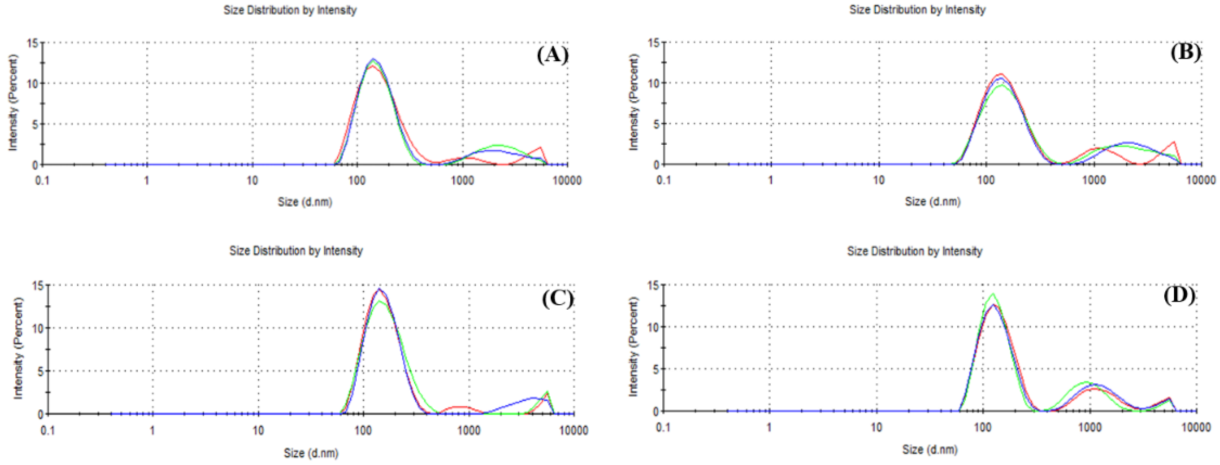

Figure S7. DLS particle size distribution profiles of DPPC/chol liposomes containing 0 and 20 mol% **IL**, hydrated with PBS (pH 7.4) and measured after 7 days of storage. (**A**) DPPC/chol liposomes containing 0 mol% **IL**, stored at 4 °C; (**B**) DPPC/chol liposomes containing 20 mol% **IL**, stored at 4 °C; (**C**) DPPC/chol liposomes containing 0 mol% **IL**, stored at RT; (**D**) DPPC/chol liposomes containing 20 mol% **IL**, stored at RT. "0" means DPPC/chol liposomes without **IL**. Three replicate measurements are shown for each formulation (red - replicate 1; green - replicate 2; blue - replicate 3).

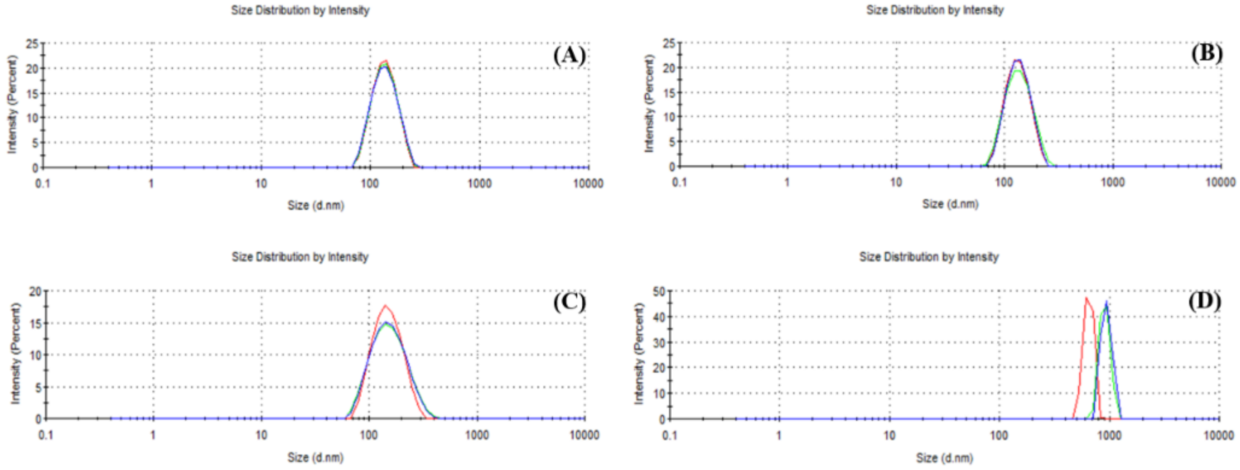

Figure S8. DLS particle size distribution profiles of DPPC/chol liposomes containing 0 and 20 mol% **IL**, hydrated with milli-Q water and measured after 14 days of storage. (**A**) DPPC/chol liposomes containing 0 mol% **IL**, stored at 4 °C; (**B**) DPPC/chol liposomes containing 20 mol% **IL**, stored at 4 °C; (**C**) DPPC/chol liposomes containing 0 mol% **IL**, stored at RT; (**D**) DPPC/chol liposomes containing 20 mol% **IL**, stored at RT. "0" means DPPC/chol liposomes without **IL**. Three replicate measurements are shown for each formulation (red - replicate 1; green - replicate 2; blue - replicate 3).

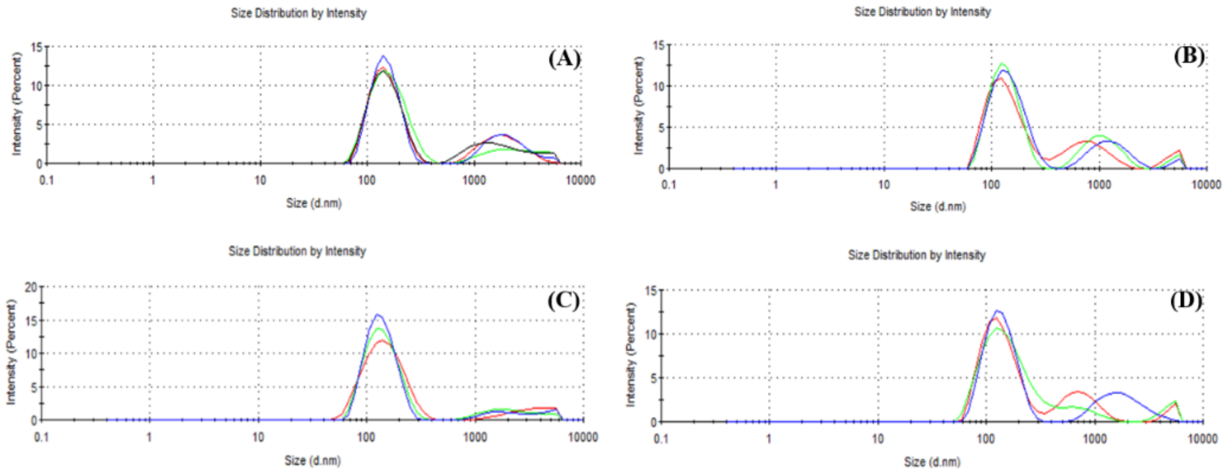

Figure S9. DLS particle size distribution profiles of DPPC/chol liposomes containing 0 and 20 mol% **IL**, hydrated with PBS (pH 7.4) and measured after 14 days of storage. (**A**) DPPC/chol liposomes containing 0 mol% **IL**, stored at 4 °C; (**B**) DPPC/chol liposomes containing 20 mol% **IL**, stored at 4 °C; (**C**) DPPC/chol liposomes containing 0 mol% **IL**, stored at RT; (**D**) DPPC/chol liposomes containing 20 mol% **IL**, stored at RT. "0" means DPPC/chol liposomes without **IL**. Three replicate measurements are shown for each formulation (red - replicate 1; green - replicate 2; blue - replicate 3).

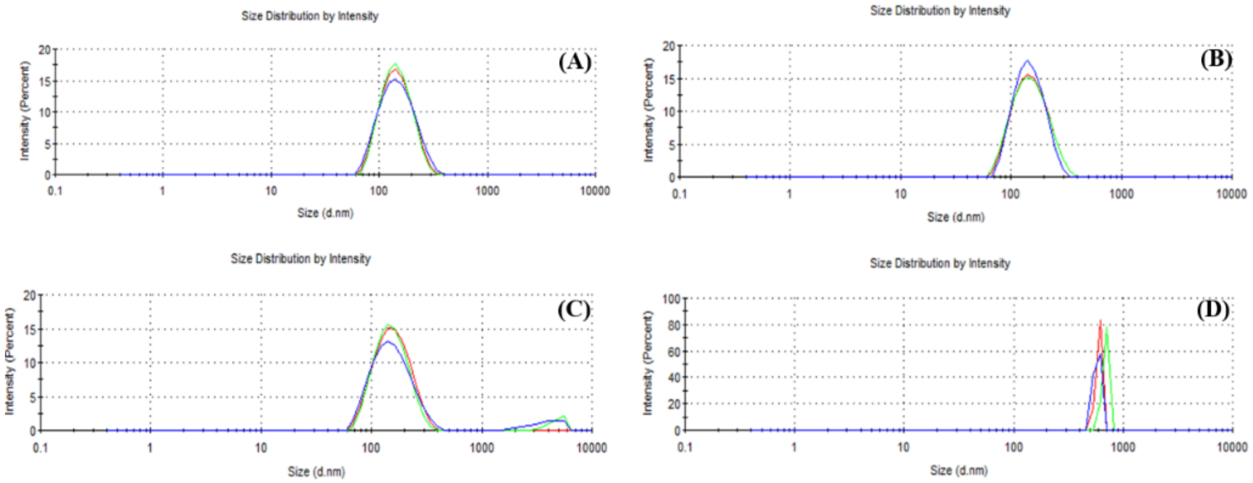

Figure S10. DLS particle size distribution profiles of DPPC/chol liposomes containing 0 and 20 mol% **IL**, hydrated with milli-Q water and measured after one month of storage. (**A**) DPPC/chol liposomes containing 0 mol% **IL**, stored at 4 °C; (**B**) DPPC/chol liposomes containing 20 mol% **IL**, stored at 4 °C; (**C**) DPPC/chol liposomes containing 0 mol% **IL**, stored at RT; (**D**) DPPC/chol liposomes containing 20 mol% **IL**, stored at RT. "0" means DPPC/chol liposomes without **IL**. Three replicate measurements are shown for each formulation (red - replicate 1; green - replicate 2; blue - replicate 3).

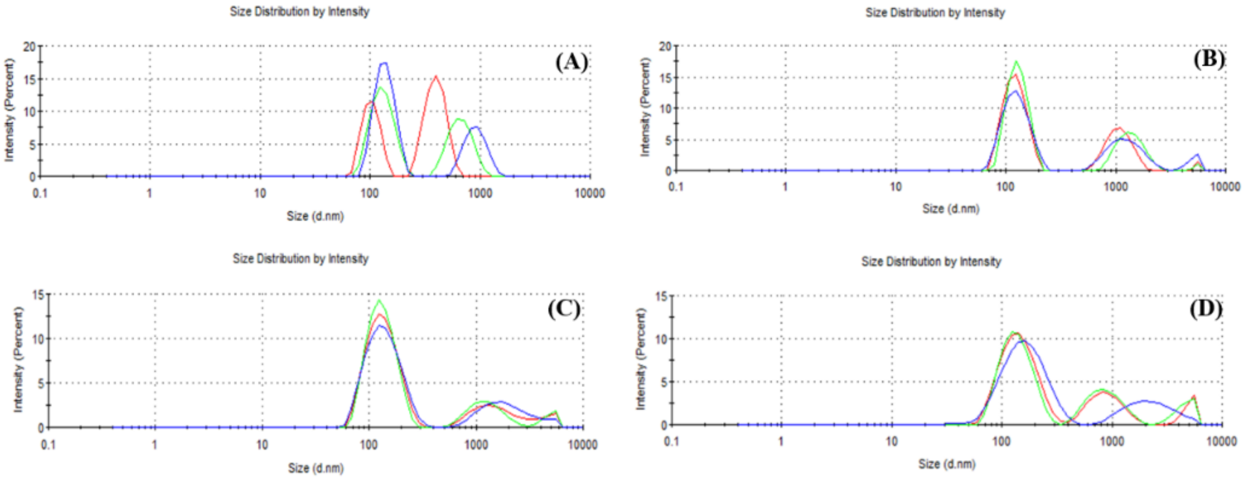

Figure S11. DLS particle size distribution profiles of DPPC/chol liposomes containing 0 and 20 mol% **IL**, hydrated with PBS (pH 7.4) and measured after one month of storage. (**A**) DPPC/chol liposomes containing 0 mol% **IL**, stored at 4 °C; (**B**) DPPC/chol liposomes containing 20 mol% **IL**, stored at 4 °C; (**C**) DPPC/chol liposomes containing 0 mol% **IL**, stored at RT; (**D**) DPPC/chol liposomes containing 20 mol% **IL**, stored at RT. "0" means DPPC/chol liposomes without **IL**. Three replicate measurements are shown for each formulation (red - replicate 1; green - replicate 2; blue - replicate 3).

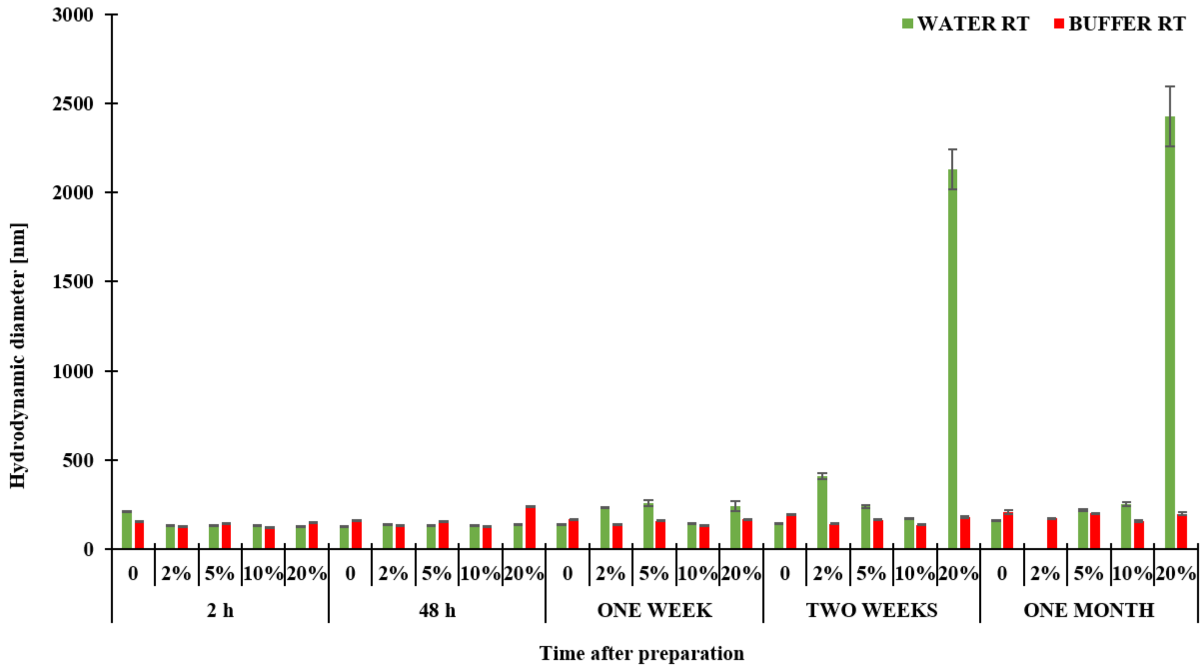

Figure S12. Hydrodynamic diameter of DPPC/chol liposomes containing 0, 2, 5, 10, and 20 mol% **IL**, measured after preparation (2 h) and after 48 h, one week, two weeks, and one month of storage. "0" means DPPC/chol liposomes without **IL**. Data are presented as mean ± standard deviation ($n = 3$).

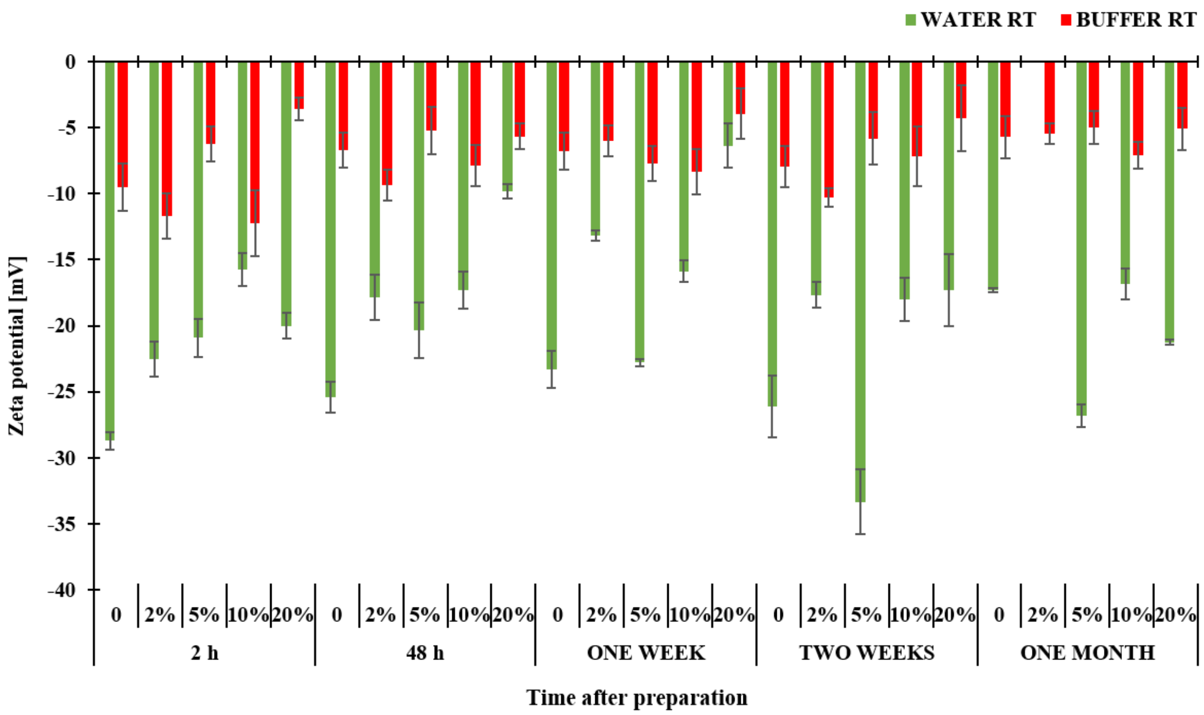

Figure S13. Zeta potential of DPPC/chol liposomes containing 0, 2, 5, 10, and 20 mol% **IL**, measured after preparation (2 h) and after 48 h, one week, two weeks, and one month of storage. "0" means DPPC/chol liposomes without **IL**. Data are presented as mean ± standard deviation (n = 3).

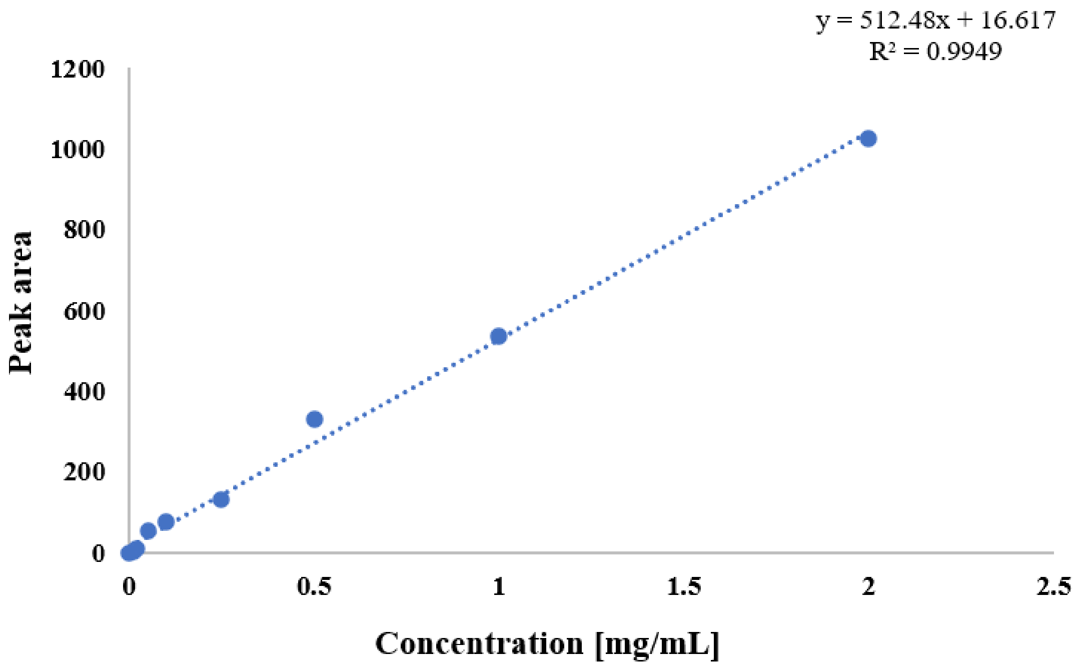

Figure S14. Calibration curve for the quantitative determination **IL** by GC. The curve was obtained using **IL** standard solutions in the concentration range of 0-2 mg/mL.

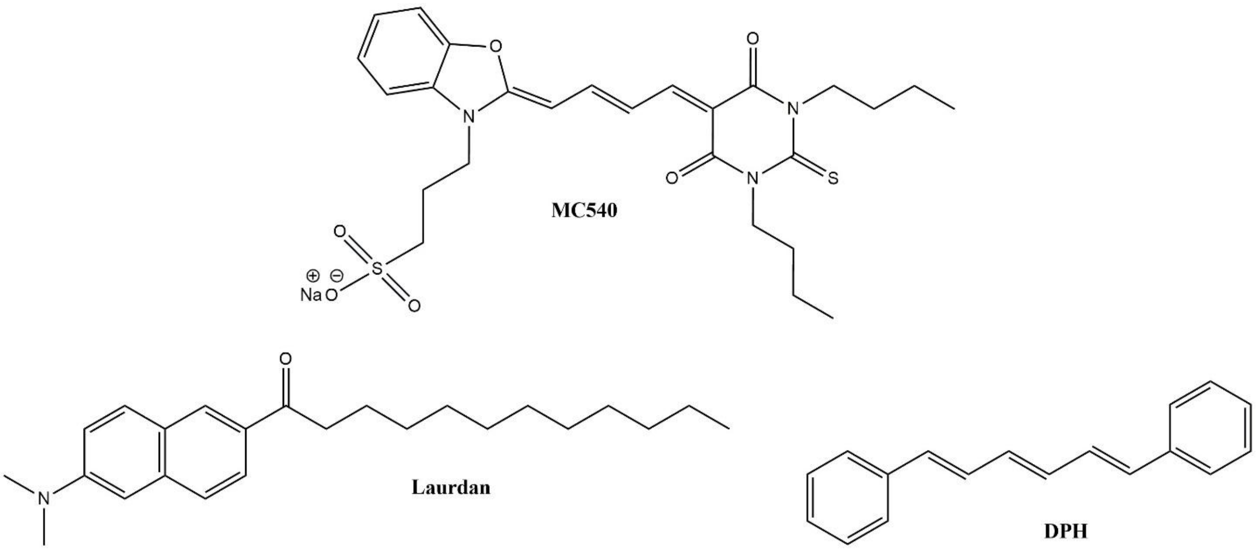

Figure S15. Chemical structures of merocyanine 540 (MC540), 1-[6-(dimethylamino)naphthalen-2-yl]dodecan-1-one (Laurdan), and 1,6-diphenyl-1,3,5-hexatriene (DPH) used as fluorescent membrane probes.

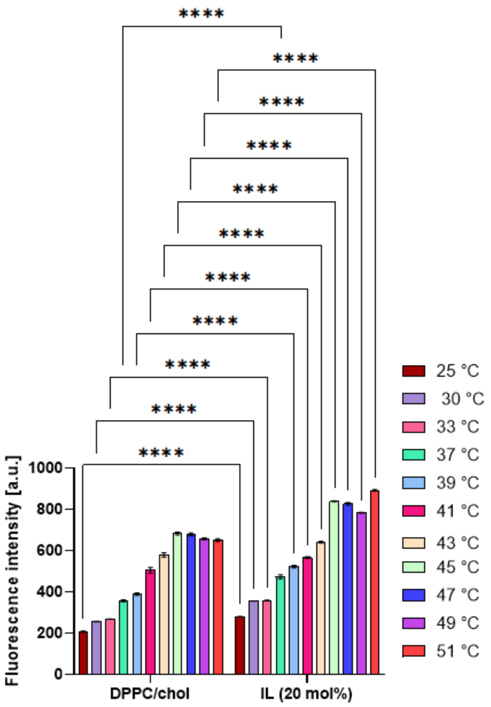

Figure S16. Statistical analysis of the data presented in **Figure 5**. Statistical analysis was performed using two-way analysis of variance (ANOVA), followed by Šídák's multiple comparisons test. Data are presented as mean ± standard deviation (n = 3).

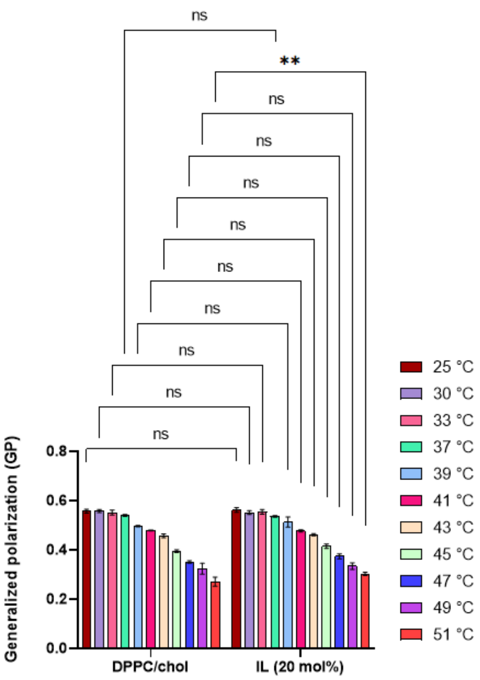

Figure S17. Statistical analysis of the data presented in **Figure 6**. Statistical analysis was performed using two-way analysis of variance (ANOVA), followed by Šídák's multiple comparisons test. Data are presented as mean ± standard deviation (n = 3).

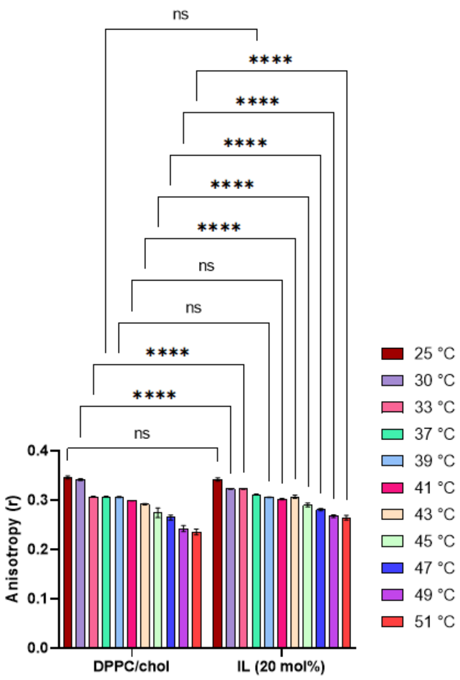

Figure S18. Statistical analysis of the data presented in **Figure 7**. Statistical analysis was performed using two-way analysis of variance (ANOVA), followed by Šídák's multiple comparisons test. Data are presented as mean ± standard deviation (n = 3).

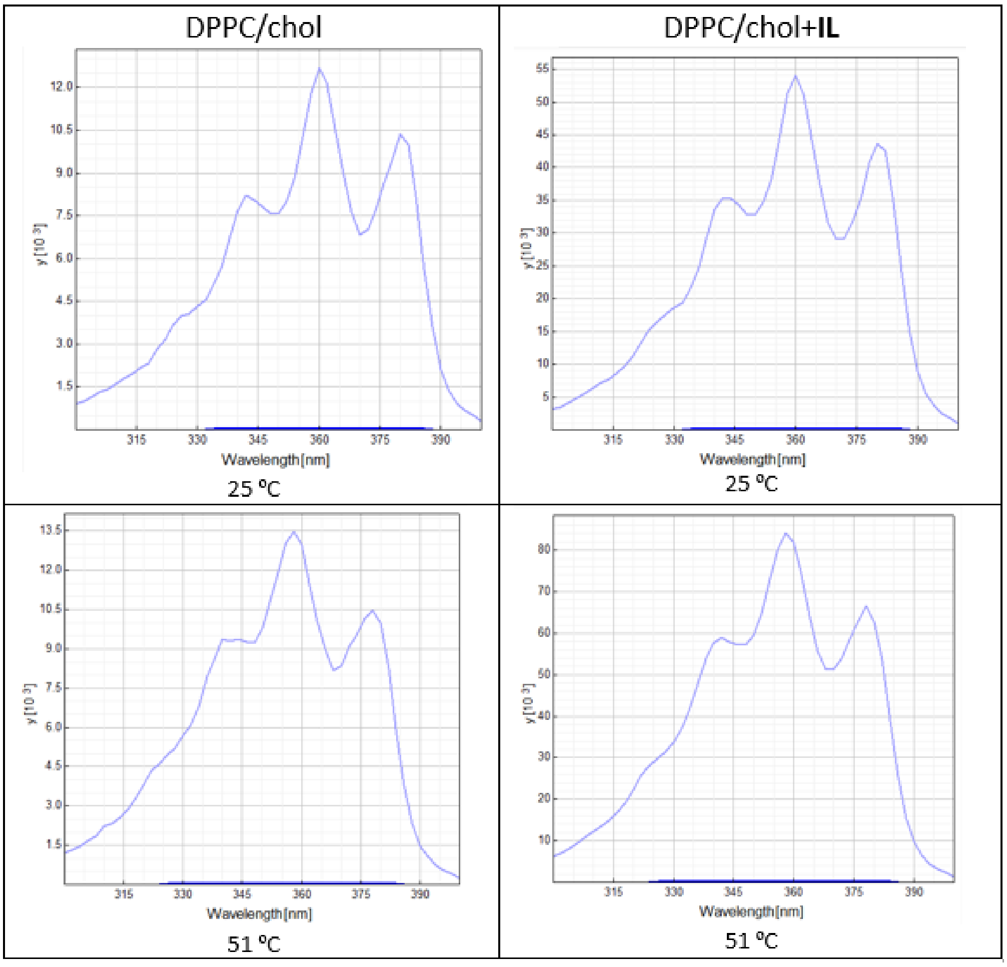

Figure S19. Raw fluorescence excitation spectra of the DPH recorded at two temperatures for DPPC/chol liposomes and DPPC/chol liposomes with the addition of 20 mol% **IL**.

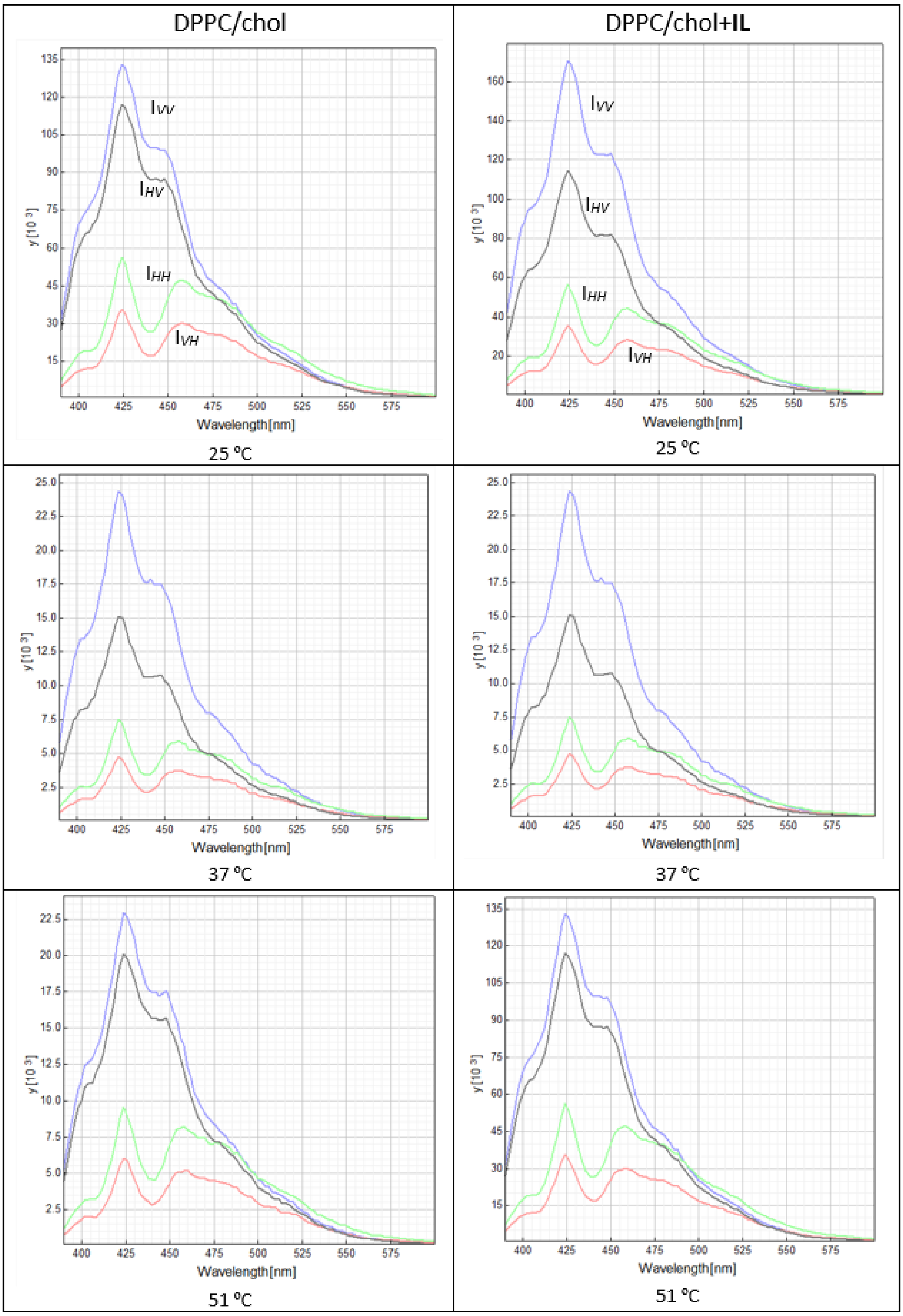

Figure S20. Raw polarized fluorescence intensity components spectra (*I_{VV}*, *I_{VH}*, *I_{HV}*, *I_{HH}*) of the DPH probe recorded at three temperatures for: pure liposomes DPPC/chol and liposomes with the addition of 20 mol% **IL**. ***I_{VV}*** - vertically polarized excitation/vertically polarized emission; ***I_{VH}*** - vertically polarized excitation/horizontally polarized emission; ***I_{HV}*** - horizontally polarized excitation/vertically polarized emission; ***I_{HH}*** - horizontally polarized excitation/horizontally polarized emission.

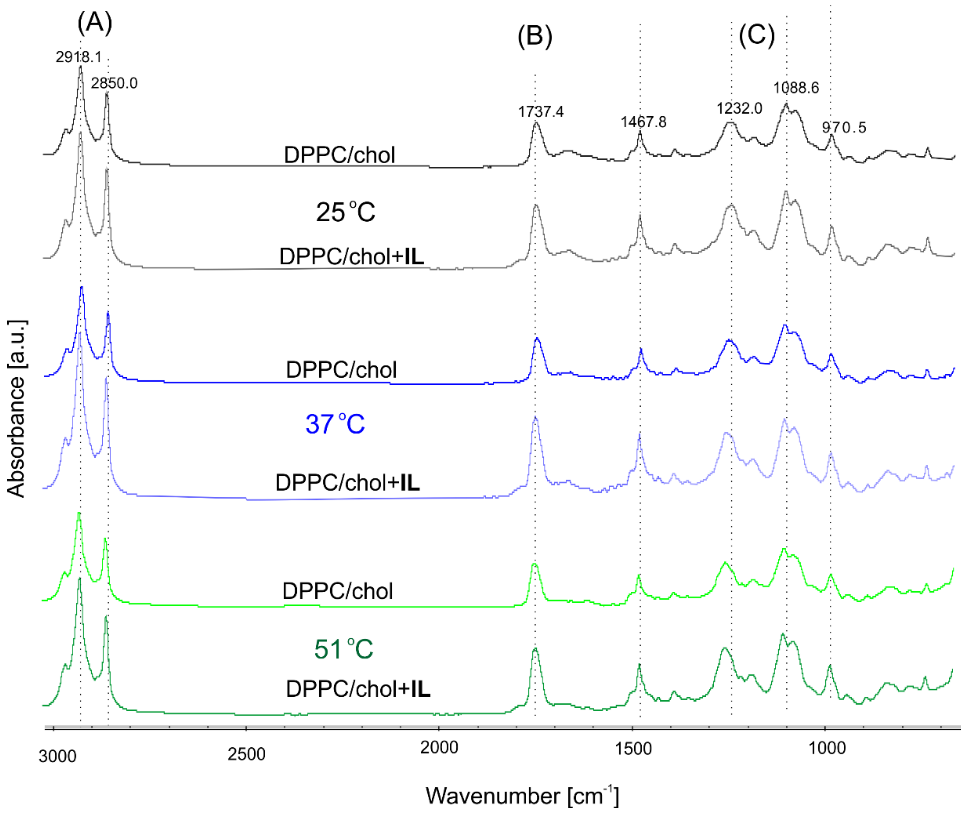

Figure S21. ATR-FTIR spectra of dehydrated DPPC/chol liposomes and liposomes in the presence of **IL** (20 mol%) at different temperatures. The main diagnostic regions are indicated: (**A**) C-H stretching vibrations of the hydrocarbon chains (2800-3000 cm^{-1}), (**B**) carbonyl group stretching vibrations (1700-1750 cm^{-1}), and (**C**) phosphate group vibrations (1080-1260 cm^{-1}).

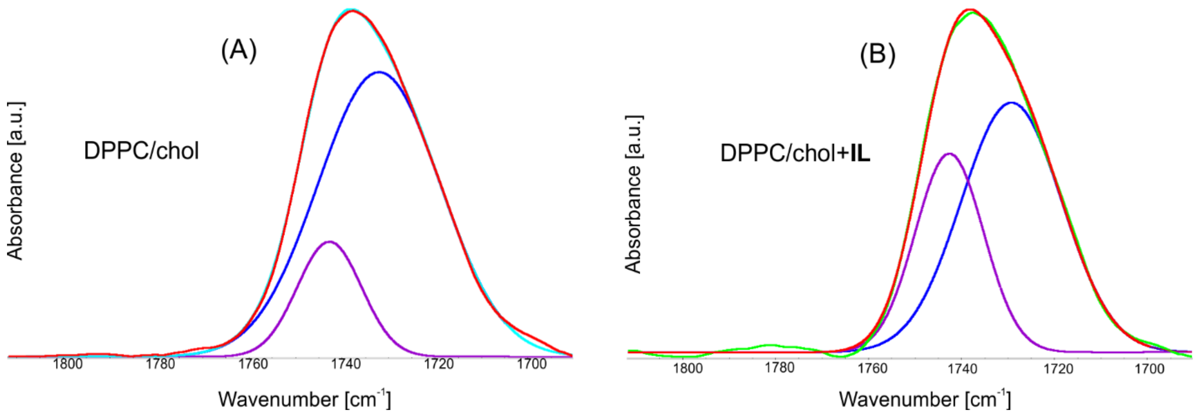

Figure S22. Deconvolution of the carbonyl group (C=O) stretching vibration band in the ATR-FTIR spectrum measured at 25 °C for: (**A**) DPPC/chol liposomes and (**B**) liposomes with the addition of 20 mol% **IL**. Line designations: green/cyan - experimental spectrum, red - fitted cumulative curve, purple - component of free C=O groups (~1742 cm^{-1}); blue - component of hydrogen-bonded C=O groups (~1730 cm^{-1}).

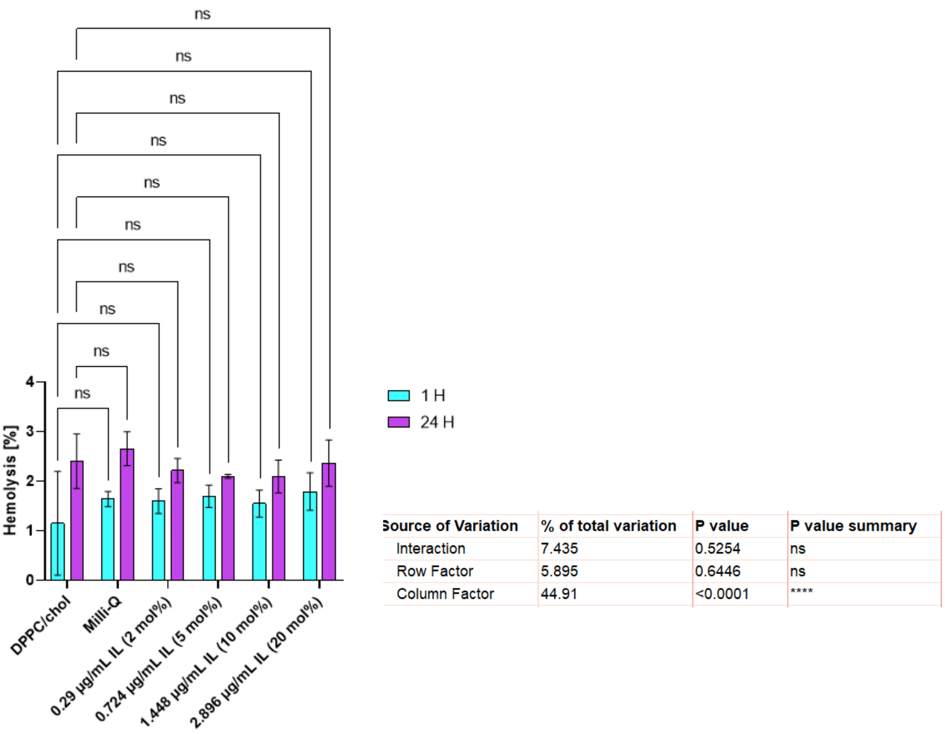

Source of Variation	% of total variation	P value	P value summary
Interaction	7.435	0.5254	ns
Row Factor	5.895	0.6446	ns
Column Factor	44.91	<0.0001	****

Figure S23. Statistical analysis of the data presented in **Figure 8.** Statistical analysis was performed using two-way analysis of variance (ANOVA), followed by Dunnett's multiple comparisons test for comparisons with the control group. Data are presented as mean ± standard deviation (n = 3).

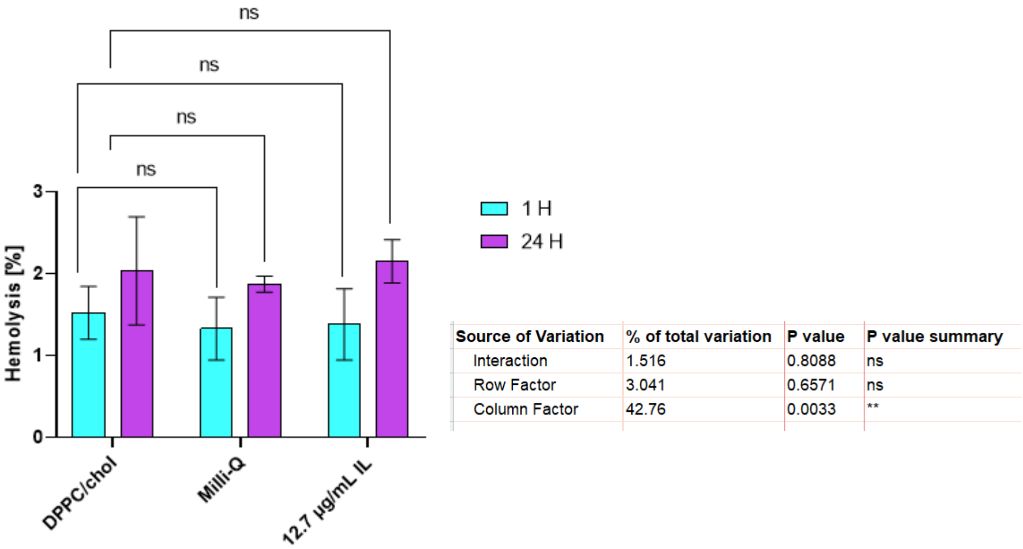

Source of Variation	% of total variation	P value	P value summary
Interaction	1.516	0.8088	ns
Row Factor	3.041	0.6571	ns
Column Factor	42.76	0.0033	**

Figure S24. Statistical analysis of the data presented in **Figure 9**. Statistical analysis was performed using two-way analysis of variance (ANOVA), followed by Dunnett's multiple comparisons test for comparisons with the control group. Data are presented as mean ± standard deviation (n = 3).
